# Temporal and Spatial Metabolic Shifts Revealing the Transition from Ulcerative Colitis to Colitis‐Associated Colorectal Cancer

**DOI:** 10.1002/advs.202412551

**Published:** 2025-01-22

**Authors:** Ruiqi Sun, Yuanyuan Zhang, Xian Zhao, Tian Tang, Yuepeng Cao, Liu Yang, Yuan Tian, Zunjian Zhang, Pei Zhang, Fengguo Xu

**Affiliations:** ^1^ Key Laboratory of Drug Quality Control and Pharmacovigilance (Ministry of Education), State Key Laboratory of Natural Medicines China Pharmaceutical University Nanjing 210009 P. R. China; ^2^ Department of Pharmacy Drum Tower Hospital China Pharmaceutical University Nanjing 210008 P. R. China; ^3^ School of Pharmacy Air Force Medical University Xi'an 710032 P. R. China; ^4^ The Affiliated Cancer Hospital of Nanjing Medical University Jiangsu Cancer Hospital Jiangsu Institute of Cancer Research Nanjing 210009 P. R. China

**Keywords:** colitis‐associated colorectal cancer (CAC), eicosapentaenoic acid (EPA), insulin‐like growth factor binding protein 5 (IGFBP5), metabolomics, ulcerative colitis (UC)

## Abstract

Patients with ulcerative colitis (UC) have a higher risk of developing colorectal cancer (CRC), however, the metabolic shifts during the UC‐to‐CRC transition remain elusive. In this study, an AOM‐DSS‐induced three‐stage colitis‐associated colorectal cancer (CAC) model is constructed and targeted metabolomics analysis and pathway enrichment are performed, uncovering the metabolic changes in this transition. Spatial metabolic trajectories in the “normal‐to‐normal adjacent tissue (NAT)‐to‐tumor” transition, and temporal metabolic trajectories in the “colitis‐to‐dysplasia‐to‐carcinoma” transition are identified through K‐means clustering of 74 spatially and 77 temporally differential metabolites, respectively. The findings reveal two distinct metabolic profile categories during the inflammation‐to‐cancer progression: those with consistent changes, either increasing (e.g., kynurenic acid, xanthurenic acid) or decreasing (e.g., long‐chain fatty acids, LCFAs), and those enriched at specific disease stages (e.g., serotonin). Further analysis of metabolites with consistent temporal trends identifies eicosapentaenoic acid (EPA) as a key metabolite, potentially exerting anti‐inflammatory and anti‐cancer effects by inhibiting insulin‐like growth factor binding protein 5 (IGFBP5). This study reveals novel metabolic mechanisms underlying the transition from UC to CAC and suggests potential targets to delay the progression.

## Graphical abstract

In the ulcerative colitis to colitis‐associated colorectal cancer transition, metabolic changes vary at specific disease stages. Normal adjacent tissue is metabolically distinct from both normal and tumor tissues. Eicosapentaenoic acid is a key metabolite exerting anti‐inflammatory and anti‐cancer effects by inhibiting insulin‐like growth factor binding protein 5.

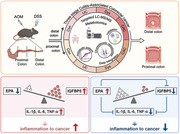

## 1 Introduction

Colorectal cancer (CRC) is the third most prevalent cancer and the leading cause of cancer‐related deaths worldwide.^[^ ^1^ ^]^ Individuals with long‐term inflammatory bowel disease (IBD), especially ulcerative colitis (UC), have a 2–3 times higher risk of developing colitis‐associated colorectal cancer (CAC) compared to the general population, with CAC accounting for 10–15% of IBD‐related deaths annually.^[^ ^2^, ^3^, ^4^ ^]^ CAC typically arises in the distal colon, corresponding to the area of active inflammation in IBD.^[^ ^5^, ^6^ ^]^ Moreover, normal adjacent tissue (NAT), which differs in gene expression and metabolic activity, may contribute to tumorigenesis and progression.^[^ ^7^ ^]^ Notably, CAC follows a temporal progression marked by “colitis‐to‐dysplasia‐to‐carcinoma” and tends to advance more rapidly,^[^ ^8^ ^]^ exhibits greater resistance to therapeutic interventions, and results in higher mortality compared to sporadic CRC.^[^ ^9^, ^10^, ^11^ ^]^ Therefore, early detection and intervention to prevent or delay the UC‐to‐CRC transition is critical. However, the underlying mechanism that drives the transition remains elusive.

Efforts to investigate the molecular mechanisms of inflammation‐to‐cancer transition have employed multi‐omics strategies, with most studies analyzing inflammation and tumor samples separately. Microbiomics suggests that bacteria such as *Escherichia coli* and Enterotoxigenic *Trichoderma fragilis* play roles in the development of IBD and CRC.^[^ ^12^, ^13^, ^14^, ^15^, ^16^ ^]^ Transcriptomic studies reveal that dysregulation of the Wnt pathway biases CAC toward mesenchymal tumor subtypes, potentially affecting prognosis and treatment options.^[^ ^17^, ^18^ ^]^ Metabolomics, targeted sequencing of cancer‐related genes, transcriptomics, and methylated DNA immunoprecipitation sequencing show that the proto‐oncogene protein MYC regulates overall metabolic reprogramming in CRC by modulating 215 metabolic reactions.^[^ ^19^ ^]^ However, this fragmented approach fails to capture the dynamic evolution of the disease. A few studies examine continuous disease processes, such as transcriptomic and epigenomic analyses of a CAC mouse model, revealing the dynamic chromatin changes during the inflammation‐to‐cancer transition.^[^ ^20^ ^]^ While transcriptomics and epigenomics provide insights into gene regulation and activity, they do not directly correlate with functional outcomes or final phenotypes.^[^ ^21^ ^]^ Moreover, existing studies overlook the dysplastic stage of CAC progression and fail to reflect the dynamic temporal and spatial metabolic switch linking inflammation to cancer.

Metabolic reprogramming emerges as a critical mechanistic link that orchestrates the progressive transformation from inflammatory colonic tissues to malignant states. Elucidating these metabolic alterations is essential for identifying early intervention targets and developing preventive strategies for CAC. Metabolomics complements these studies by providing a direct perspective on biological metabolism, reflecting the functional consequences of gene expression changes, and contributing to a more comprehensive understanding of disease mechanisms.^[^ ^22^, ^23^ ^]^ Metabolomics has proven valuable for studying progressive diseases. For instance, large‐scale metabolomic analyses of clear cell renal cell carcinoma have identified elevated glutathione and dipeptide levels during clinical progression.^[^ ^24^ ^]^ Similarly, targeted metabolomic analysis of invasive lung adenocarcinomas and their precursors has mapped metabolic trajectories from atypical adenomatous hyperplasia to invasive adenocarcinoma, revealing abnormalities in bile acid metabolism that may serve as potential therapeutic targets.^[^ ^25^ ^]^

Recent studies have provided important insights into metabolic regulation in colonic diseases. Yu et al. (2024) profiled 1251 subjects to reveal characteristic plasma and fecal metabolic signatures during CRC progression (normal‐adenoma‐CRC), demonstrating how distinct metabolites significantly influence disease progression through specific signaling pathways.^[^ ^26^ ^]^ Their findings underscore the crucial role of metabolic regulation in colonic tissue transformation. Additionally, spatial metabolic analyses have revealed important insights. Jain et al. (2024) demonstrated significant metabolic gradients in inflammatory colonic tissues across 372 patients,^[^ ^27^ ^]^ while Ikuta et al. (2024) showed that non‐tumor areas in CRC patients exhibited altered gene expression compared to healthy individuals, particularly in advanced cases.^[^ ^28^ ^]^ These findings collectively highlight the importance of spatial metabolic variation in disease progression. However, a systematic investigation of dynamic metabolic alterations during CAC development remains lacking, particularly regarding the regulatory mechanisms underlying spatio‐temporal metabolic reprogramming during disease progression. Moreover, while studies have demonstrated substantial metabolic heterogeneity within colonic inflammatory tissues, the patterns and characteristics of these spatial metabolic variations during the UC‐to‐CRC transition require comprehensive investigation.

In this study, we implemented an integrated spatio‐temporal metabolomics analysis strategy to systematically investigate the metabolic dynamics during colitis‐cancer transformation. We employed a targeted quantitative metabolomics approach to investigate the temporal and spatial metabolic changes associated with inflammation‐to‐cancer transition using a three‐stage CAC model induced by AOM‐DSS. Spatial metabolic changes were characterized by analyzing differential metabolite change trends in normal tissues, NAT, and tumor tissues at the CAC stage. Temporal metabolic changes were assessed by examining metabolite change trends across the UC, dysplasia (Dys), and CAC stages. Additionally, the mechanisms of action of the identified key metabolites during the inflammation‐to‐cancer transition were further explored.

## 2 Results

### 2.1 UC‐CAC Animal Model Construction

**Figure 1:**
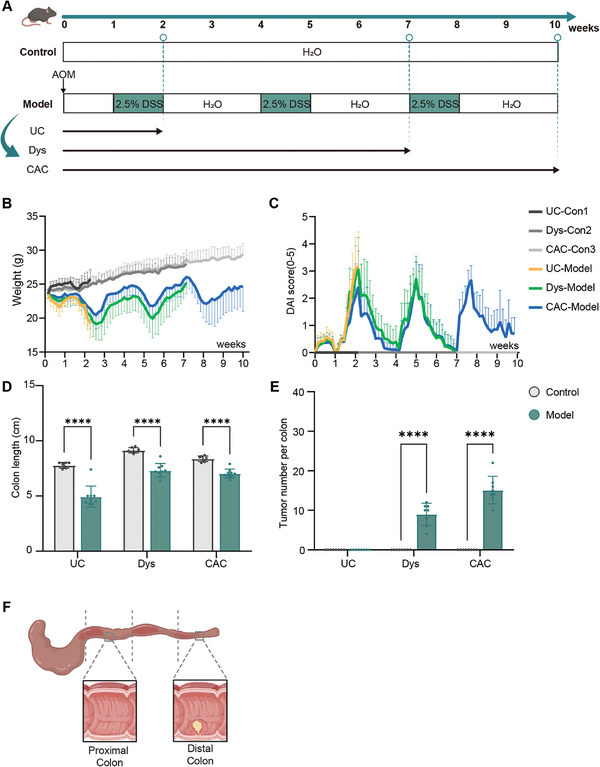
Construction of a multi‐stage CAC model. A) Workflow illustrating the metabolomics landscape study of a three‐stage CAC model. Mice were intraperitoneally injected with AOM (10 mg kg^−1^), followed by 2.5% DSS in drinking water. Tissues were collected and analyzed at the indicated time points, with 8 biological replicates for each time point. B) Measurement of mouse body weight during the experimental period (n = 8). C) Assessment of DAI during the experimental period (n = 8). D) Colon length of mice from control, UC, Dys, and CAC groups (n = 8). E) Division of the colon into the proximal and distal colon segments. F) Number of colorectal tumors per mouse (n = 8). All data are expressed as means ± SD. Statistical significance was determined using an unpaired two‐tailed Student's *t*‐test (D, F). ****p < 0.0001.

To explore the metabolic changes over time and space during the transition from inflammation to cancer in CAC, we induced a three‐stage CAC mouse model using azoxymethane‐dextran sulfate sodium salt (AOM‐DSS) (**Figure** **1**A). Specifically, mice received a single intraperitoneal injection of AOM (12.5 mg kg^−1^), followed by three cycles of 2.5% DSS in drinking water (7 days on, 14 days off). Colon and tumor tissues were collected at weeks 2, 7, and 10 post‐AOM injections, representing UC, Dys, and CAC stages, respectively.

The AOM‐DSS model demonstrated consistent pathological progression with distinct stage‐specific characteristics. After each round of DSS feeding, mice exhibited significant weight loss (Figure 1B), accompanied by increased disease activity index (DAI) (Figure 1C), and a marked reduction in colon length (Figure 1D; Figure S1A, Supporting Information). Disease progression showed characteristic features at each stage: week 2 captured the UC stage with extensive immune cell infiltration and mucosal damage, week 7 represented the emergence of dysplastic lesions with architectural distortion of colonic crypts, and week 10 demonstrated the CAC stage with invasive growth patterns. The number of distal tumors increased progressively, reaching 100% incidence by week 10 (Figure 1E; Figure S1B,C, Supporting Information). A survival rate of 80% was maintained throughout the experiment, with a humane endpoint being reached at a weight loss of 15–20%.

Spatially, tumor formation displayed distinct spatial specificity. Only the distal colon exhibited beaded tumor aggregates during the CAC stage, distinguishing the distal (tumor tissue) from the proximal colon (NAT), allowing for spatial metabolomics analysis (Figure 1F; Figure S1B,C, Supporting Information).

### 2.2 Spatial Metabolite Alterations at the CAC Stage

**Figure 2:**
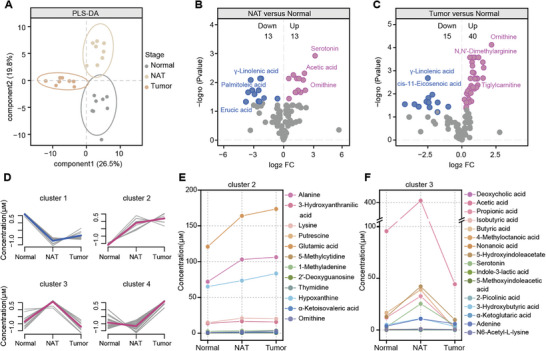
Spatial metabolite alterations at the CAC stage. A) PLS‐DA score plot showing metabolic characteristics of different spatial sites. Gray represents the normal group, light yellow represents NAT (proximal colon site), and yellow represents the tumor group (distal colon site). B,C) Volcano plots showing differential metabolites in NAT versus normal tissue (B) and tumor tissue versus normal tissue (C). Significant changes were defined using the criteria of FC (fold change) ≥ 1.5 or FC ≤ 0.667, and *p* < 0.05. Metabolites significantly increased or decreased are indicated in pink and blue, respectively. D) Metabolic trajectory clustering using differential metabolites from normal, NAT, and tumor tissues. E,F) Dynamic changes of metabolites in clusters 2 and 3. Each point represents a metabolite.

Previous studies on metabolic alterations of CRC have predominantly focused on analyzing tumor tissue and paired NAT.^[^ ^29^ ^]^ However, recent findings suggest that NAT represents a distinct state between normal and tumor tissues, which may exhibit unique metabolic alterations.^[^ ^7^, ^28^, ^30^, ^31^ ^]^ A targeted liquid chromatography‐mass spectrometry (LC‐MS)‐based metabolomics method developed previously was applied in this study to capture the metabolic changes during UC‐CRC progression. This method applies chemical derivatization and is characterized by relatively high coverage and good sensitivity. With this method, we compared the metabolic profiles of normal, NAT, and tumor tissues at the CAC stage. Partial least squares discriminant analysis (PLS‐DA) clearly distinguished three groups (**Figure** **2**A). Compared to the normal tissue group, the NAT and tumor tissue exhibited 26 and 55 differential metabolites, respectively (Figure 2B,C). Moreover, 55 differential metabolites were identified between NAT and tumor tissues (Figure S2A, Supporting Information).

To investigate the spatial characteristics of differential metabolites, a total of 74 unique differential metabolites obtained from the above comparisons were subjected to K‐means cluster analysis. Four distinct clusters of metabolites were identified (Figure 2D). Among the consistently changing clusters, cluster 1, primarily composed of long‐chain fatty acids (LCFAs), showed a significant down‐regulation from normal to NAT, with minimal changes observed between NAT and tumor tissues (Figure S2B, Supporting Information). Conversely, cluster 2 showed consistent up‐regulation, including metabolites like ornithine (Orn) (Figure 2E). The other clusters exhibited significant alterations at specific stages: cluster 3 was up‐regulated only in NAT (e.g., serotonin), while cluster 4 showed significant up‐regulation exclusively in tumor tissues (e.g., tryptophan and proline) (Figure 2F; Figure S2C, Supporting Information).

In summary, our findings reveal that NAT represents a metabolically distinct state, distinguishing it from normal and tumor tissues. Further research on NAT could help elucidate the transition from inflammation to cancer and provide opportunities for early tumor intervention and the discovery of new therapeutic targets.

### 2.3 Temporal Metabolic Changes in the Proximal Colon during the Colitis‐To‐Dysplasia‐To‐Carcinoma Transition

**Figure 3:**
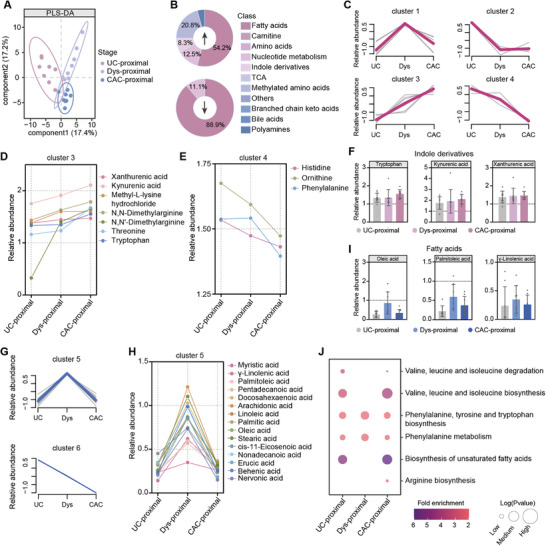
Temporal metabolic changes in the proximal colon from colitis‐to‐dysplasia‐to‐carcinoma. A) PLS‐DA score plot of the proximal colon at UC, Dys, and CAC stages. Pink represents the UC stage, light blue represents the Dys stage, and blue represents the CAC stage. B) Percentage of different types of differential metabolites detected in the proximal colon. C) Metabolic trajectory clustering of up‐regulated differential metabolites in the proximal colon. D,E) Dynamic changes of metabolites in clusters 3 and 4. Each point represents a metabolite. F) Histograms of representative metabolites in clusters 3 and 4, showing the change trend in the relative abundance of metabolites. G) Metabolic trajectory clustering of down‐regulated differential metabolites in the proximal colon. H) Dynamic changes of metabolites in cluster 5. Each point represents a metabolite. I) Histograms of representative metabolites in cluster 5. J) Kyoto Encyclopedia of Genomes (KEGG) metabolic pathways altered in the proximal colon during the UC, Dys, and CAC stages compared to their corresponding controls.

Histologic inflammation in the proximal colon increases the risk of CAC in patients with IBD, especially those with primary sclerosing cholangitis.^[^ ^32^ ^]^ Therefore, studying metabolic alterations in the proximal colon could provide insights into the mechanisms underlying inflammation to cancer transformation. To investigate these metabolic changes, we analyzed alterations of the proximal colon at different stages, comparing them to corresponding controls. Principal component analysis (PCA) score plots revealed significant metabolic changes across all stages (Figure S3A–C, Supporting Information). PLS‐DA further distinguished the proximal colon of the UC, Dys, and CAC stages (**Figure** **3**A).

To elucidate the trajectory of metabolite changes during the colitis‐to‐dysplasia‐to‐carcinoma transition in the proximal colon, we first categorized the differential metabolites based on their changing trends and then performed K‐means clustering analysis (Figure 3B). Four distinct clusters were identified among the 24 up‐regulated metabolites (Figure 3C). Some metabolites exhibited significant up‐regulation only at specific stages. For instance, metabolites in cluster 1 were significantly up‐regulated only at the Dys stage, while those in cluster 2 were enriched predominantly at the UC stage, such as putrescine (Figure S3D,E, Supporting Information). Additionally, many metabolites displayed consistent changes over time, such as those in clusters 3 and 4. In particular, cluster 3 metabolites (e.g., kynurenic acid, xanthurenic acid, tryptophan) showed a steady increase in up‐regulation throughout the inflammation‐to‐cancer transformation (Figure 3D), whereas the up‐regulation of cluster 4 metabolites (e.g., Orn) diminished over time (Figure 3E). Of particular note, tryptophan and its downstream metabolites, including kynurenine and xanthurenic acid, showed a gradual increase (Figure 3F). Similarly, two clusters were identified among the 18 down‐regulated metabolites (Figure 3G). Cluster 5, primarily composed of LCFAs, displayed significant down‐regulation from colitis‐to‐dysplasia‐to‐carcinoma (Figure 3H,I). The degree of down‐regulation of cluster 6 metabolites increased throughout the inflammation‐to‐cancer transformation (Figure S3F, Supporting Information).

Next, we conducted enrichment analyses of differential metabolites obtained from each stage. Certain metabolic pathways were significantly altered only at specific stages, such as arginine biosynthesis, which was altered only at the CAC stage (Figure 3J). In contrast, some metabolic alterations appeared to persist over time, particularly at the Dys stage. For example, the phenylalanine, tyrosine, and tryptophan biosynthesis, along with phenylalanine metabolism, exhibited the greatest alterations at the Dys stage, showing a pattern of initial increase followed by a decrease (Figure 3J). On the other hand, pathways related to valine, leucine, and isoleucine synthesis and degradation, as well as unsaturated fatty acid biosynthesis, remained unchanged at the Dys stage but were significantly altered at both the UC and CAC stages, suggesting a potential stage‐specific metabolic regulation in the proximal colon during the Dys stage (Figure 3J).

Overall, these findings outline the trajectory of metabolic alterations from UC to CAC in the proximal colon, highlighting consistent changes in metabolites and metabolic pathways associated with disease progression. Notably, stage‐specific metabolic regulation during the Dys stage was observed, suggesting the presence of distinct metabolic phenotypes at this critical phase of disease development.

### 2.4 Temporal Metabolic Changes in the Distal Colon during the Colitis‐To‐Dysplasia‐To‐Carcinoma Transition

**Figure 4:**
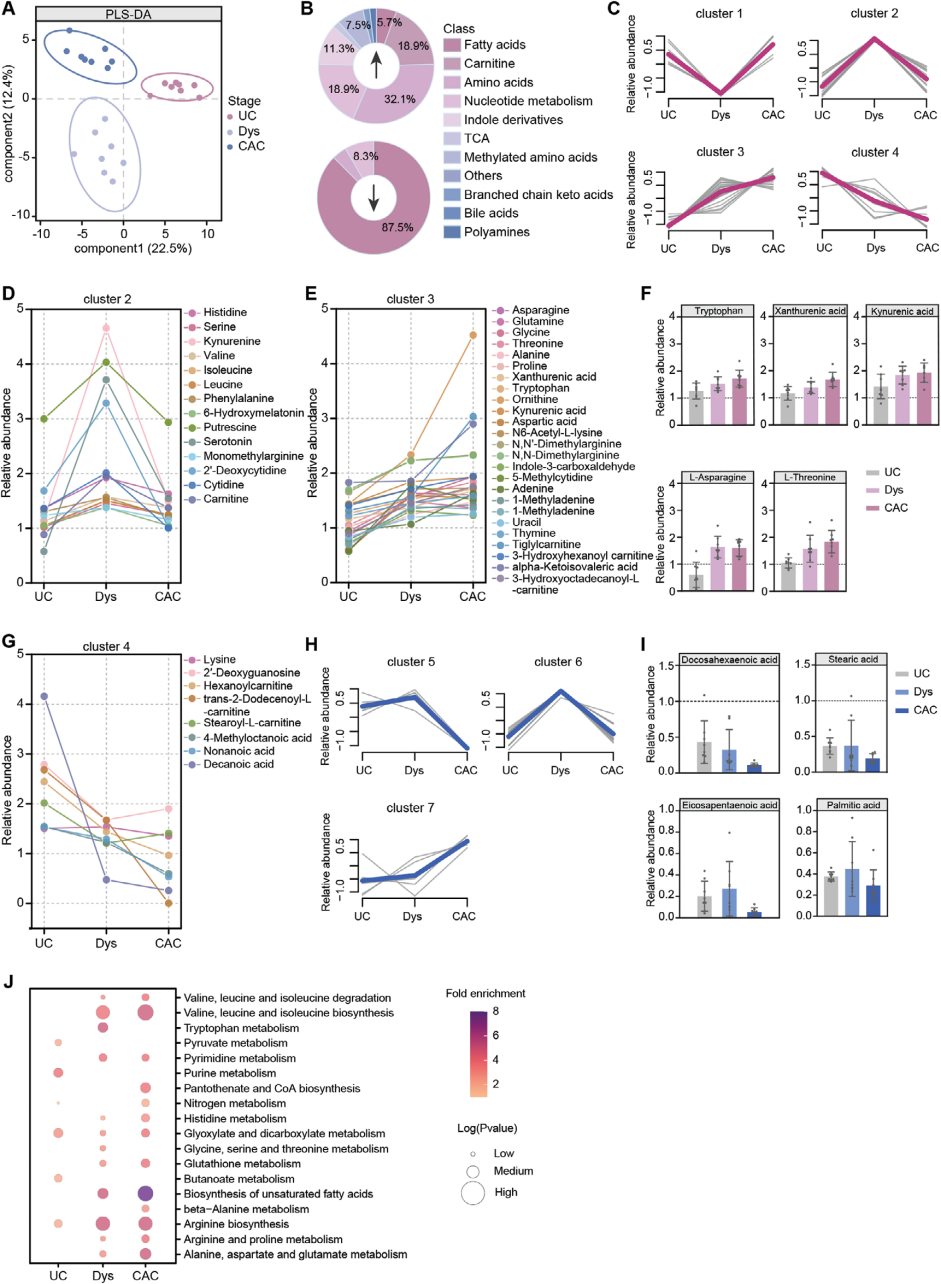
Temporal metabolic changes in the distal colon from colitis to dysplasia to carcinoma. A) PLS‐DA score plot of the distal colon at the UC, Dys, and CAC stages. Pink represents the UC stage, light blue represents the Dys stage, and blue rerepsents the CAC stage. B) Percentage of different types of differential metabolites detected in the distal colon. C) Metabolic trajectory clustering of up‐regulated differential metabolites in the distal colon. D,E) Dynamic changes of metabolites in clusters 2 and 3. Each point represents a metabolite. F) Histograms of representative metabolites in cluster 3, indicate the change trend in relative abundance of metabolites. G) Histograms of representative metabolites in cluster 4. Each point represents a metabolite. H) Metabolic trajectory clustering of down‐regulated differential metabolites in the distal colon. I) Dynamic changes of metabolites in cluster 5, showing the trend of change in relative abundance of metabolites. J) KEGG metabolic pathways altered in the distal colon during the UC, Dys, and CAC stages compared to their corresponding controls.

UC typically affects the rectum and spreads continuously from the distal to the proximal colon.^[^ ^33^ ^]^ The distal colon experiences prolonged inflammation compared to the proximal colon, with more extensive foci of isodysplasia.^[^ ^34^ ^]^ Given the pivotal role of metabolic reprogramming in cancer initiation and progression,^[^ ^22^, ^35^ ^]^ we examined metabolic alterations at the tumorigenesis site (distal colon) compared to corresponding controls at each stage. PCA score plots revealed significant metabolic changes across all stages (Figure S4A–C, Supporting Information). The PLS‐DA score plot separated the distal colon at the UC, Dys, and CAC stages (**Figure** **4**A). To investigate the trajectory of metabolite changes in the distal colon, we categorized differential metabolites at these stages based on their regulation trends and performed a K‐means clustering analysis (Figure 4B).

We identified four clusters of 53 up‐regulated metabolites (Figure 4C). Certain clusters, such as clusters 1 and 2, were significantly up‐regulated only at specific stages (Figure 4D; Figure S4D, Supporting Information). In particular, cluster 2 metabolites, including kynurenine and 2′‐deoxycytidine, were enriched at the Dys stage, suggesting their potential role in tumor promotion (Figure 4D). Conversely, some metabolites showed consistent changes over time, such as those in clusters 3 and 4. Cluster 3 metabolites, including Orn, kynurenic acid, and xanthurenic acid, steadily increased throughout the colitis‐to‐dysplasia‐to‐carcinoma transformation (Figure 4E). Interestingly, indole derivatives like tryptophan, kynurenic acid, and xanthurenic acid, as well as threonine and asparagine, demonstrated similar changing trends in both the proximal and distal colon (Figure 4F). In contrast, cluster 4 metabolites showed a decreasing trend over the entire period, as exemplified by decanoic acid (C10) (Figure 4G).

Among the 24 down‐regulated metabolites, we identified three clusters, predominantly composed of LCFAs, with clusters 5 or 7 exhibiting a gradual increase or decrease in down‐regulation over time (Figure 4H,I; Figure S4E–G, Supporting Information). Furthermore, certain metabolites displayed opposing changes across different disease stages. For example, glutamate, hypoxanthine, 3‐hydroxy dodecanoyl carnitine, and 3‐hydroxyanthranilic acid showed contrasting alterations from colitis‐to‐dysplasia‐to‐carcinoma (Figure S4H, Supporting Information).

Enrichment analysis was conducted to explore the metabolic pathway alterations in the distal colon. Some metabolic pathways were significantly altered only at specific stages (Figure 4J). For instance, pyruvate metabolism, purine metabolism, and butyrate metabolism were affected only at the UC stage, while tryptophan metabolism, glycine, serine, and threonine metabolism were impacted solely at the Dys stage. Similarly, pantothenic acid, CoA biosynthesis, and beta‐alanine metabolism were influenced only at the CAC stage. Conversely, some pathways showed consistent changes over time, such as arginine biosynthesis, which was significantly up‐regulated across all three stages (Figure 4J). Pathways related to the synthesis and degradation of valine, leucine and isoleucine, histidine metabolism, glutathione metabolism, unsaturated fatty acid biosynthesis, arginine and proline metabolism, and alanine, aspartate, and glutamate metabolism also showed a gradual increase in Dys stage and CAC stage (Figure 4J).

While similar temporal metabolomic patterns were observed in both the proximal and distal colon from UC to CAC, the trends in metabolite alterations were either aligned or opposite in the distal colon. As the distal colon is a tumor‐prone site, the modulation of metabolites that undergo consistent changes in the early stages of the disease (e.g., Orn, eicosapentaenoic acid (EPA)) may play a crucial role in the inflammation‐to‐cancer transformation process. Notably, metabolic alteration trends in time are similar to space, with mutations present in the intermediate Dys stage, and modulation of mutant metabolites specific to this stage may affect inflammation‐to‐cancer transformation.

### 2.5 EPA Exerts both Anti‐Inflammatory and Anti‐Cancer Effects

Next, we aimed to explore the biological functions of metabolites that displayed consistent changing trends in the distal colon across different stages. Among the up‐regulated metabolites, Orn showed a consistent increase, with significant changes at the CAC stage. C10 exhibited a consistent down‐regulation trend, with the most significant changes at the UC stage. EPA also displayed a consistent decrease, with significant changes during the Dys and CAC stages (Figure S5A, Supporting Information). Therefore, we further investigated the potential role of C10, Orn, and EPA in the inflammation‐to‐cancer transformation.

**Figure 5:**
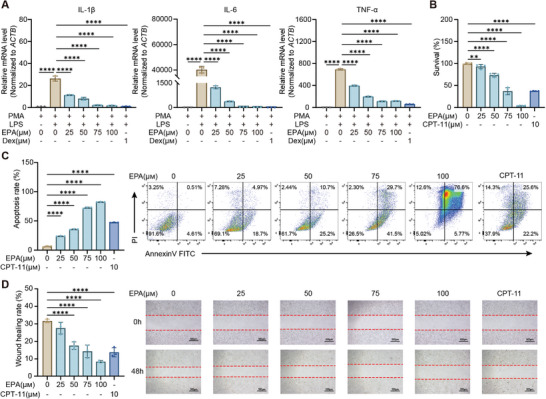
EPA exerts anti‐inflammatory and anti‐cancer effects. A) RT‐qPCR analysis of TNF‐α, IL‐1β, and IL‐6 in LPS‐induced THP‐1 macrophages treated with EPA and Dex (n = 3). B) Impact of EPA and CPT‐11 treatment on cell proliferation in HCT116 cells (n = 3). C) Effects of EPA and CPT‐11 treatment on apoptosis of HCT116 cells (n = 3). D) Scratch assay of HCT116 cells treated with EPA and CPT‐11 after 0 and 48 hours of incubation (n = 3). All data are expressed as means ± SD. Statistical significance was determined using one‐way ANOVA with Bonferroni's correction (A–D). *p < 0.05, **p < 0.01, ***p < 0.001, ****p < 0.0001.

In the LPS‐stimulated THP‐1 inflammatory cell model, EPA, Orn, and C10 suppressed the mRNA levels of pro‐inflammatory cytokines IL‐1β, IL‐6, and TNF‐α, with EPA showing the most pronounced dose‐dependent inhibitory effect comparable to the positive control dexamethasone (Dex) (**Figure** **5**A; Figure S5B, Supporting Information). Moreover, EPA, Orn, and C10 demonstrated significant antitumor activity, with EPA exhibiting the most potent effect comparable to the positive control irinotecan (CPT‐11). At 75 µm, EPA exhibited antineoplastic efficacy equivalent to CPT‐11, significantly suppressing cell proliferation and migration while inducing apoptosis, outperforming both Orn and C10 (Figure 5B–D; Figure S6A–C, Supporting Information). Further studies on colonic epithelial cells showed that EPA inhibited IL‐1β‐induced elevation of ICAM‐1, a marker of endothelial damage, in CCD841 CoN in a dose‐dependent manner (Figure S6D, Supporting Information). Notably, the median concentration of EPA measured in the mouse colon was 130.17 µm, which is consistent with the concentration range of EPA used in our cell experiments. Taken together, these results reveal that EPA possesses the most pronounced anti‐inflammatory and anti‐cancer effects compared to Orn and C10.

### 2.6 EPA Exerts Anti‐Inflammatory and Anti‐Cancer Effects via IGFBP5

**Figure 6:**
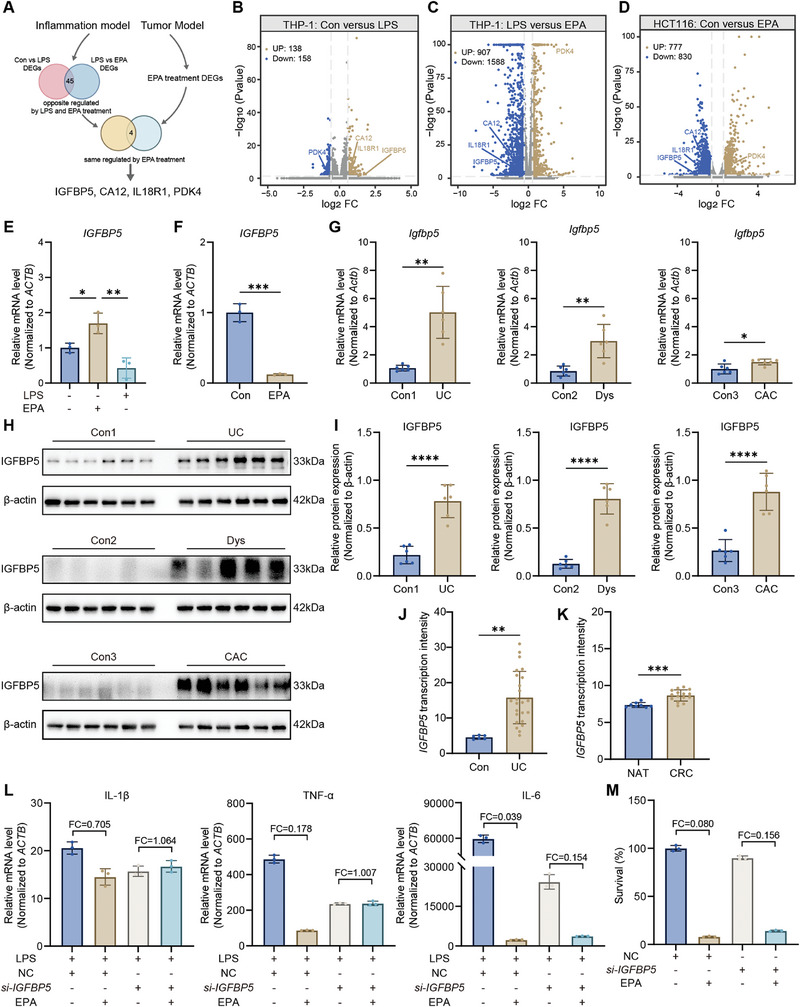
Transcriptomics analysis of the mechanism of EPA. A) Schematic representation of the transcriptome data analysis strategy. B–D) Volcano plots illustrating the FC and *p* value of differentially expressed genes (DEGs): (B) LPS‐induced inflammation group versus control in THP‐1 macrophages, (C) EPA‐treated group versus LPS‐induced inflammation group in THP‐1 macrophages, and (D) EPA‐treated group versus control in HCT116 cells. Significant changes were defined using FC ≥ 1.5 or FC ≤ 0.667, and *p* < 0.05. Yellow dots indicate upregulated genes, blue dots indicate downregulated genes, and grey dots represent genes with no significant change. E) RT‐qPCR analysis of *IGFBP5* expression in LPS‐induced THP‐1 macrophages treated with EPA (75 µm). F) RT‐qPCR analysis of *IGFBP5* expression in HCT116 cells treated with EPA (75 µm). G) RT‐qPCR analysis of *Igfbp5* expression in the distal colon animal at different stages. H,I) Western blot analysis of IGFBP5 expression in the distal colon of animals at different disease stages. J) Gene expression analysis of *IGFBP5* in UC from the GSE16879 RNA‐Seq dataset. K) Gene expression analysis of *IGFBP5* in CRC from the GSE4183 RNA‐Seq dataset. L) IGFBP5 knockdown attenuated the effects of EPA (75 µm) on inflammatory gene expression (TNF‐α, IL‐6, and IL‐1β) in LPS‐induced THP‐1 macrophages. M) The impact of EPA (75 µm) treatment on cell proliferation in IGFBP5‐knockdown HCT116 cells. All data are expressed as means ± SD. Statistical significance was determined using an unpaired two‐tailed Student's *t*‐test (E–G, I–K) or one‐way ANOVA with Bonferroni's correction (L, M). *p < 0.05, **p < 0.01, ***p < 0.001, ****p < 0.0001.

To further elucidate the molecular mechanisms underlying EPA‐regulated inflammation and tumor processes, we performed transcriptome sequencing after treating LPS‐induced THP‐1 inflammatory cells and the HCT116 tumor cells with EPA (75 µm) for 48 h. The PCA score plot showed a clear separation between the control, LPS group, and LPS+EPA group in THP‐1 cells (Figure S7A, Supporting Information). Similarly, the control and EPA groups were clearly separated in HCT116 cells (Figure S7B, Supporting Information). Using the criteria of FC ≥ 1.5 or ≤ 0.667 and *p* < 0.05, we identified differential genes that were reversed after LPS induction and EPA treatment in the inflammatory cell model, as well as genes significantly altered by EPA treatment in the tumor cell model. Venn diagram analysis revealed four genes that were significantly altered in both models: insulin‐like growth factor binding protein 5 (IGFBP5), carbonic anhydrase 12 (CA12), interleukin 18 receptor 1 (IL18R1), and pyruvate dehydrogenase kinase 4 (PDK4) (**Figure** **6**A–D).

To verify the reliability of the transcriptome sequencing results, we first examined the expression changes of these differential genes in the cell models by RT‐qPCR. The results showed that EPA treatment significantly altered the relative mRNA levels of *IGFBP5*, *CA12*, *IL18R1*, and *PDK4* in both THP‐1 cells and HCT116 cells, with the trends of change being consistent with the sequencing data (Figure 6E,F; Figure S7C,D, Supporting Information). Further validation in AOM‐DSS animal models revealed that only the relative mRNA level of *IGFBP5* was consistent with the results from the cell models (Figure 6G; Figure S7E, Supporting Information). Western blot analysis showed that IGFBP5 protein was significantly up‐regulated at all disease stages in the AOM‐DSS‐induced CAC model (Figure 6H,I). Additionally, data from GEO databases showed that *IGFBP5* levels were significantly up‐regulated in both UC and CRC tissues (Figure 6J,K).

To investigate the role of IGFBP5 in the biological functions of EPA, we used siRNA to knock down IGFBP5 expression in THP‐1 and HCT116 cells. The results showed that the inhibitory effects of EPA on inflammatory factors were altered after IGFBP5 knockdown: the inhibitory effect on TNF‐α was completely abolished, the effect on IL‐6 was partially retained, and the inhibitory effect on IL‐1β was significantly weakened (Figure 6L). Moreover, the inhibitory effect of EPA on tumor cell proliferation was also significantly decreased (Figure 6M). These findings suggest that IGFBP5 is an important molecular target through which EPA exerts its anti‐inflammatory and anti‐cancer effects.

## 3 Discussion

The mechanisms underlying the transformation of UC to CAC remain unclear, posing a challenge for current CAC therapies.^[^ ^6^ ^]^ Previous studies have typically analyzed UC and CRC separately, suggesting that consistently altered metabolites during the inflammation‐cancer transition contribute to tumorigenesis.^[^ ^18^ ^]^ However, these studies overlook the Dys stage in the UC‐to‐CAC progression, failing to capture the dynamic changes in disease phenotype. In this study, we conducted a targeted metabolomic analysis of an AOM‐DSS‐induced CAC model across three stages – UC, Dys, and CAC, which enabled us to systematically map both temporal (colitis‐to‐dysplasia‐to‐carcinoma) and spatial (normal‐to‐NAT‐to‐tumor) metabolic shifts during the inflammation‐to‐cancer progression.

During disease progression, we observed that LCFAs, especially EPA, were the most down‐regulated metabolites at the Dys and CAC stages. While we are reporting for the first time that EPA undergoes significant changes in UC‐to‐CAC transformation, existing literature has documented changes in EPA levels in UC or CRC. In UC, significant EPA depletion has been consistently documented in pathological tissues through both colonic mucosal biopsy analysis and case‐control investigations.^[^ ^36^, ^37^ ^]^ In the context of CRC, large‐scale epidemiological investigations have established an inverse correlation between EPA levels and CRC risk, characterized by both diminished serum EPA concentrations in CRC patients and significant protective associations in cohort analyses.^[^ ^38^, ^39^, ^40^ ^]^ Moreover, studies revealed significantly lower EPA levels in cancerous tissue compared to normal tissue,^[^ ^41^ ^]^ with more pronounced EPA depletion in metastatic versus non‐metastatic CRC specimens.^[^ ^42^, ^43^ ^]^ The decline in EPA levels from inflammation to cancer could potentially be attributed to an imbalance in synthesis and metabolic regulation. In terms of synthesis, inflammatory factors such as TNF‐α and IL‐6 suppress the expression of fatty acid desaturase 2 in a time‐dependent manner, thereby hindering *de novo* EPA production.^[^ ^44^, ^45^ ^]^ On the metabolic side, the upregulation of cyclooxygenases, lipoxygenases, and cytochrome P450 enzyme systems promotes the conversion of EPA into its downstream metabolites.^[^ ^46^, ^47^, ^48^ ^]^ Additionally, disease‐associated oxidative stress exacerbates EPA decline through non‐enzymatic oxidative degradation.^[^ ^49^ ^]^

EPA has demonstrated significant effects in disease treatment. In UC models, EPA shows remarkable therapeutic efficacy by alleviating tissue damage and reducing inflammation through multiple mechanisms.^[^ ^50^, ^51^, ^52^, ^53^ ^]^ Furthermore, studies in cancer models have revealed that EPA effectively suppresses tumor progression in various CRC models.^[^ ^54^, ^55^, ^56^, ^57^ ^]^ Clinical studies have validated these experimental findings, demonstrating that EPA‐FFA administration effectively reduces inflammatory markers in UC patients and improves outcomes in CRC patients.^[^ ^52^, ^58^, ^59^ ^]^ The safety and long‐term efficacy of EPA supplementation have been well‐documented through extensive clinical studies. Meta‐analyses of 83 randomized controlled trials (>41000 participants) confirmed that EPA significantly reduced both inflammatory bowel disease recurrence and disease progression.^[^ ^60^ ^]^ Long‐term studies demonstrated that EPA‐FFA administration (2 g day^−1^) achieved an 89.5% therapeutic response rate in UC patients and showed significant chemopreventive activity against colorectal adenomas.^[^ ^52^, ^61^ ^]^ The American Heart Association and the European Medicines Agency have recognized its safety for clinical use.^[^ ^62^, ^63^ ^]^ Based on our findings, supported by the literature discussed above, it is believed that EPA holds great potential in the treatment of CAC.

From the perspective of temporal metabolic shifts, we also uncovered a stage‐specific pattern of metabolite changes during the transformation from inflammation to cancer for the first time. Among these, metabolites such as serotonin and putrescine were significantly enriched at the dysplastic stage. Putrescine has been reported to alleviate inflammation through intestinal epithelial renewal and promote anti‐inflammatory macrophage activity,^[^ ^64^ ^]^ while stimulating colonic tumor cell proliferation.^[^ ^65^, ^66^ ^]^ Similarly, previous studies have highlighted serotonin's dual role in both inhibiting intestinal inflammation through regulatory B cell formation and promoting cancer progression via the NLRP3 signaling pathway.^[^ ^28^, ^67^ ^]^ Our findings reconcile these seemingly contradictory functions by considering them within the context of disease progression, suggesting that these metabolites may serve as key regulators in the inflammation‐to‐cancer transition.

Spatially, CAC progresses gradually from the distal to the proximal colon, with tumor cells remodeling the surrounding stroma into a tumor microenvironment (TME). This TME provides oxygen and nutrients to meet the heightened metabolic demands of the tumor cells.^[^ ^68^ ^]^ NAT can be considered precancerous, and investigating its metabolic alteration during the CAC stage offers valuable insights into the inflammation‐to‐cancer transition. In our spatial metabolic characterization, we observed that Orn consistently increased, showing the most significant variation. Orn is an important intermediate in the urea cycle, which has been reported to be involved in multiple diseases when dysregulated.^[^ ^69^, ^70^ ^]^ For example, ornithine decarboxylase (ODC) plays a role in autoimmune colitis by influencing the activity of group 3 innate lymphoid cells in the intestine, with its activity progressively increasing in cancer cells.^[^ ^71^, ^72^ ^]^ Additionally, pancreatic cancer cells enhance polyamine synthesis via ornithine aminotransferase, contributing to tumor growth.^[^ ^73^ ^]^ During inflammation‐cancer transformation, the significant elevation of Orn levels reflects a severe disruption in metabolic homeostasis. Pro‐inflammatory cytokines, such as TNF‐α and IL‐1β, upregulate Arginase 1 (Arg1) expression within the inflammatory microenvironment, driving the conversion of arginine to Orn.^[^ ^74^, ^75^, ^76^ ^]^ Chronic inflammation sustains elevated Arg1 activity, resulting in Orn accumulation. Furthermore, infiltrating macrophages and neutrophils express Arg1, creating a positive feedback loop between the release of inflammatory factors and Arg1 activation.^[^ ^77^ ^]^ The elevated tissue Orn subsequently supports tumor cell proliferation via ODC‐mediated polyamine synthesis, perpetuating a vicious cycle that accelerates disease progression.^[^ ^78^ ^]^

Despite these findings, there are limitations that should be addressed in future studies. First, efforts should be directed toward validating these results in human UC‐CRC samples and additional validated models to further establish their clinical relevance and strengthen the connection between preclinical discoveries and clinical applications. Second, while our study highlights the role of IGFBP5 in EPA's biological functions, the precise mechanisms by which EPA metabolites regulate IGFBP5 expression remain unclear. Further experiments are needed to determine whether EPA influences the translation or degradation of IGFBP5. Third, the use of nontargeted metabolomics platforms capable of capturing novel metabolite features could provide more comprehensive profiling in future studies, potentially uncovering previously uncharacterized metabolites that may play crucial roles in the inflammation‐to‐cancer transition.

Overall, our study utilized a targeted metabolomics approach to analyze AOM‐DSS‐induced CAC model mice across three disease stages, uncovering the metabolic trajectories of inflammation‐to‐cancer transition over time and space. We identified distinct alterations in metabolites at each stage of this transformation process, with some metabolic changes showing consistent increases or decreases, while others were specifically enriched at particular disease stages. Notably, our findings suggest that EPA may exert anti‐inflammatory and anti‐cancer effects, highlighting its therapeutic potential in alleviating the progression from UC to CAC. These insights could improve early clinical diagnosis, offering new opportunities for accelerating accurate detection and intervention at early stages of the disease.

## 4 Experimental Section

### Chemicals and Reagents

Azoxymethane (AOM, CAS: 25843‐45‐2) was purchased from Sigma‐Aldrich (USA), and dextran sulfate sodium salt (DSS, CAS: 02160110‐CF) was purchased from MP Biomedicals. The inventory of standards of metabolites encompassing 9 classes (acylcarnitines, polyamines, amino acids, methylated amino acids, branched chain keto acids, fatty acids, indole derivatives, nucleotide metabolism, tricarboxylic acid cycle) is provided in Table S1 (Supporting Information). Further details on materials and reagents used for metabolomic analysis are available in the Experimental Procedures section of the Supporting Information.

### Experimental Animals

SPF‐grade male C57BL/6 mice (22–24 g) were purchased from Vital River Laboratory Animal Technology Co., Ltd. (Beijing, China) and acclimated for one week. All animal experiments followed the protocol approved by the Animal Ethics Committee of China Pharmaceutical University (No: 2022‐06‐025).

### CAC Mice Model Establishment

The AOM/DSS‐induced mouse CAC model was established as described previously.^[^ ^79^, ^80^ ^]^ Initially, 60 eight‐week‐old male mice were randomly assigned to three model groups (UC stage, Dys stage, CAC stage) and a control group (n = 8). Mice in the model groups received an intraperitoneal injection of AOM (10 mg kg^−1^ body weight). After 1 week, they were given 2.5% DSS (w/v) in drinking water for one week, followed by 2 weeks of regular water. This round was repeated three times, and the mice were sacrificed at designated time points. Mice in the control group were provided regular drinking water. The distinct stages of disease were sampled at three time points: 1) acute UC stage: seven days post‐DSS administration; 2) dysplasia (Dys) stage: at the end of the second round of regular water; and 3) CAC stage: at the end of the third round of regular water (Figure 1A). Body weight and disease activity index (DAI) were recorded daily. The DAI was calculated based on body weight loss, stool consistency, and visible bleeding, following previous reports.^[^ ^81^ ^]^ This experimental design, accounting for potential animal loss, ensured adequate sample size and homogeneity at each time point for statistical analysis.

### Sample Collection

After euthanasia, entire colons were excised from the mice, and colon length, tumor number, and size were measured. Colon tissues were divided into proximal and distal sections (tumor‐prone regions) for further analysis, including metabolomic assays and hematoxylin and eosin (H&E) staining.

### Histopathological Examination

Colon tissues were fixed in 10% neutral‐buffered formalin at room temperature for 24 hours, then dehydrated and embedded in paraffin, and sectioned. H&E staining was performed by Servicebio (Wuhan, China).

### Metabolite Extraction

Metabolites were extracted from colon samples using precooled acetonitrile‐methanol (1:1, v/v) at a 30‐fold volume (w/v). The mixture was homogenized and sonicated in ice water for 10 minutes, then centrifuged at 14 000 rpm, 4 °C, for 10 minutes. The supernatant was dried at 37 °C under nitrogen and stored at −80 °C. Quality control samples were prepared by pooling equal amounts from each sample to evaluate the repeatability of sample preparation and instrument stability.

### Chemical Derivatization‐Based Targeted Metabolomics

The derivatization mechanisms and methods for Dns‐Cl and Dns‐PP have been reported in the previous work.^[^ ^82^, ^83^ ^]^ Amino/phenol group metabolites were derivatized with Dns‐Cl, while carboxyl group‐containing metabolites were derivatized with Dns‐PP. Modifications to the derivatization process are detailed in the Experimental Procedures section of the Supporting Information.

LC‐MS was employed for targeted metabolite profiling. Chromatographic separation was achieved on an Agilent Zorbax Eclipse XDB‐C18 column (2.1 × 100 mm, 1.8 µm) using a Shimadzu Nexera UPLC system interfaced with an 8060 triple quadruple mass spectrometer (Shimadzu Co., Kyoto, Japan). Detailed information on instrumental conditions, columns, mobile phase composition, and MS parameters are provided in the Experimental Procedures section of the Supporting Information.

### Metabolomics Data Analysis

Chromatographic peak review and integration were performed using LabSolutions LC‐MS software version 5.53 (Shimadzu Co., Tokyo, Japan). Metabolites present in at least 80% of samples within any group were considered detectable, while those present in less than 20% were assigned a baseline value of 1000. Relative abundance, defined as the metabolite intensity ratio between model and control groups at each stage, was used for further analysis. Multivariate analyses, including PLS‐DA, were performed using SIMCA‐P (Umetrics, Umea, Sweden). The Mfuzz (v.3.0) R package was used to evaluate correlations between metabolites and disease progression.

### Cell Culture and Treatment

THP‐1, CCD841 CoN, and HCT116 cell lines were obtained from the American Type Culture Collection (Manassas, VA, USA). THP‐1 cells were cultured in Roswell Park Memorial Institute (RPMI) 1640 medium (A4192301, Gibco, USA) supplemented with 10% Fetal Bovine Serum (FBS, Cat # 10100147, Gibco, USA), 1 × HEPES buffer (PYG0019, Boster, China), and 1% Penicillin‐Streptomycin (15140‐122, Gibco, USA). CCD841 CoN and HCT116 cells were cultured in Dulbecco's Modified Eagle Medium (DMEM) (Gibco, USA) supplemented with 10% FBS and 1% Penicillin‐Streptomycin. All cells were maintained at 37 °C in a humidified atmosphere containing 5% CO_2_. THP‐1 cells were differentiated with 100 ng mL^−1^ Phorbol 12‐myristate 13‐acetate (PMA, P1585, Sigma‐Aldrich, USA) for 48 h, followed by treatment with 1 µg mL^−1^ Lipopolysaccharides (LPS, L2654, Sigma‐Aldrich, USA) in the presence or absence of EPA, Orn, and C10 for 48h. CCD841 CoN cells were differentiated with 100 ng mL^−1^ IL‐1β (Novoprotein, China) and treated with EPA for 48 hours.

### Cell Viability Assay

Cells were seeded into 96‐well plates at 5000 cells per well and incubated overnight. The medium was replaced with a culture medium containing varying concentrations of EPA, Orn, and C10 for 48 h. Then, 10 µL of CCK‐8 solution was added into each well and incubated for 1–4 h at 37 °C. Absorbance was measured at 450 nm using a microplate reader (Tecan, Mannedorf, Switzerland).

### Wound Healing Assay

Cells were seeded into 6‐well plates and cultured to 80–90% confluency. Cells were scratched using a sterile plastic pipette tip (200 µL) and washed with PBS to remove debris. After that, the medium was replaced with a culture medium containing varying concentrations of EPA, Orn, and C10. Wounded areas were imaged after 0 h and 48 h of incubation and measured using ImageJ software. The data were quantified as follows: cell migration percentage = (original scratch area – new scratch area) / original scratch area.

### Apoptosis Assay

Cell apoptosis was measured using FITC‐Annexin V/PI Apoptosis kit (KeyGEN BioTECH, China). Briefly, cells were seeded into 6‐well plates and cultured to 50–60% confluency. The medium was replaced with a culture medium containing varying concentrations of EPA, Orn, and C10 for 48h. The treated cells were stained according to the manufacturer's instructions and were detected on a Beckman Flow CytoFLEX cytometer. Data were analyzed by FlowJo 10.10.0.

### Cell Transfection

The *si‐IGFBP5* and corresponding controls (NC) were purchased from Genepharma (Shanghai, China). The inserted sequence was confirmed by DNA sequencing. Transfection was performed with Hieff Trans Liposomal Transfection Reagent (40802ES, Yesen, China) according to the manufacturer's instructions. The final siRNA concentration was 20 nm. The *si‐IGFBP5* sequence was as follows: 5′‐GGACAAGUACGGGAUGAAGTT‐3′.

### Gene Expression Analysis on Databases

Microarray data and clinical information GSE16879 and GSE4183 were obtained from the Gene Expression Omnibus (GEO) database (http://www.ncbi.nih.gov/geo).^[^ ^84^, ^85^ ^]^ GSE16879 included six samples from healthy individuals and 24 from UC patients undergoing endoscopic biopsy. GSE4183 included 8 samples from healthy individuals and 15 from CRC patients undergoing endoscopic biopsy. Detailed information was listed in Tables S2 and S3 (Supporting Information).

### Transcriptome Sequencing and Analysis

Total RNA of THP‐1 and HCT116 cells was isolated using RNA isolater Total RNA Extraction Reagent (R401‐01, Vazym, China) according to the manufacturer's instructions. The libraries were prepared and sequenced using Illumina HiSeq Sequencing platform by Nanjing Paisennuo Gene Technology Co., Ltd. Differential expression genes were calculated by Bioconductor package DESeq2 (version 1.46.0) at a FC ≥ 1.5 or FC ≤ 0.667, *P* < 0.05.

### Western Blot Analysis

Cells and tissues were lysed by radioimmunoprecipitation buffer (RIPA, P0013B, Beyotime, China) containing 1 mmol L^−1^ phenylmethylsulfonyl fluoride (PMSF, ST506, Beyotime, China) for 10 min in an ice bath to obtain total proteins. Proteins were separated by SDS polyacrylamide gel electrophoresis (PAGE) and transferred to a polyvinylidene fluoride (PVDF) membrane. The membranes were then incubated with corresponding primary antibodies overnight at 4 °C and incubated with secondary antibodies at room temperature for 1 h. Immunoreactive bands were visualized on a Tanon 5200 chemiluminescence imaging system (Tanon Science & Technology) using an enhanced chemiluminescence (ECL) system (Millipore). Anti‐IGFBP5 (#10941S) was obtained from Cell Signaling Technology (CST, Danvers, MA). anti‐GAPDH (60004‐1‐Ig), anti‐ICAM‐1 (10831‐1‐AP), and anti‐mouse HRP conjugate IgG (H+L) (SA00001‐1) were purchased from Proteintech (Wuhan, China).

### Quantitative RT‐PCR (RT‐qPCR)

Total RNA was extracted from cells using RNA isolater Total RNA Extraction Reagent (R401‐01, Vazym, China). The RNA concentration was detected using NanoDrop 2000 (Thermo Fisher Scientific, USA). Then, 500 ng total RNA was reverse transcribed to complementary DNA (cDNA) using HiScript III RT SuperMix Kit (R222‐01, Vazym, China) according to the manufacturer's instructions. Subsequently, RT‐qPCR was performed using ChamQ SYBR qPCR Master Mix (Q411‐02, Vazyme, China) on a LightCycler 480 instrument (Roche) following the manufacturer's instructions. The relative expression levels of target genes were calculated by the 2^–ΔΔCT^ method and normalized by GAPDH in each sample. The primers used in RT‐qPCR are listed in Table S4 (Supporting Information).

### Statistical analysis

All data are expressed as means ± SD. For comparisons between two groups, unpaired two‐tailed Student's t‐test were applied. For comparisons among three groups, one‐way ANOVA with Bonferroni's correction was used. The detailed statistical analysis applied to each experiment is presented in the corresponding figure legends. Statistical significance was considered at *p* < 0.05. The “n” of experiments in the figure legends represents biological replicates. All data were analyzed using GraphPad Prism version 9.0. GraphPad Prism (version 9.0) and R (version 4.3.1) software were used for statistical analysis and data visualization.

## Conflict of Interest

The authors declare no conflict of interest.

## Author Contributions

R.S. and Y.Z. contributed equally to this work. R.S., F.X., and P.Z. designed the experiments. R.S., Y.Z., and T.T. performed the experiment and data analysis. Y.C. and L.Y. conducted some of the experiments. Y.T. assisted instrument analysis. F.X., P.Z., and Z.Z. directed the project. F.X., P.Z., and Z.Z. provided funding. R.S., X.Z., and P.Z. wrote the manuscript. All authors read and approved the final manuscript.

## Supporting information



Supporting Information

## Data Availability

The data that support the findings of this study are available from the corresponding author upon reasonable request.
