# Supplementary material for: Temporal and Spatial Metabolic Shifts Revealing the Transition from Ulcerative Colitis to Colitis‐Associated Colorectal Cancer
Source: Adv Sci (Weinh). 2025 Jan 22;12(11):2412551. doi: 10.1002/advs.202412551 (PMC11923922; doi:10.1002/advs.202412551)
Supplement: Supplementary file 1 — Supporting Information [file ADVS-12-2412551-s001.pdf]

ADVANCED SCIENCE
Open Access

Supporting Information

for *Adv. Sci.*, DOI 10.1002/advs.202412551

Temporal and Spatial Metabolic Shifts Revealing the Transition from Ulcerative Colitis to Colitis-Associated Colorectal Cancer

Ruiqi Sun, Yuanyuan Zhang, Xian Zhao, Tian Tang, Yuepeng Cao, Liu Yang, Yuan Tian, Zunjian Zhang, Pei Zhang* and Fengguo Xu**

Supporting Information

Temporal and Spatial Metabolic Shifts Revealing the Transition from Ulcerative Colitis to Colitis-Associated Colorectal Cancer

Ruiqi Sun[#], Yuanyuan Zhang[#], Xian Zhao, Tian Tang, Yuepeng Cao, Liu Yang, Yuan Tian, Zunjian Zhang, Pei Zhang*, Fengguo Xu**

* Correspondence to be addressed to:

Zhang ZJ (Tel/Fax: +86-25-83271454; Email: zzj@cpu.edu.cn)
Zhang P (Tel/Fax: +86-25-83271021; Email: peizhang@cpu.edu.cn)
Xu FG (Tel/Fax: +86-25-83271021; Email: fengguoxu@cpu.edu.cn)

[#] These authors contributed equally to this work.

Table of contents

Experimental Procedures
Figure S1. Establishment of the UC-CRC mouse model.
Figure S2. Spatially differentiated metabolites and cluster analysis.
Figure S3. Temporal metabolic changes from UC to CAC in the proximal colon.
Figure S4. Temporal metabolic changes from UC to CAC in the distal colon.
Figure S5. Investigation on the function of key metabolites including C10, Orn.
Figure S6. Investigation on the function of key metabolites including C10, Orn, and EPA.
Figure S7. Screening and validation of IGFBP5.

Table S1. List of standards and labeled analytes
Table S2. Clinicopathological characteristics of the included samples in the GSE16879 dataset
Table S3. Clinicopathological characteristics of the included samples in GSE4183 dataset
Table S4. The primers used in RT-qPCR

Experimental Procedures

Materials and Reagents

Standards of metabolites (Table S1, Supporting Information) were purchased from Sigma-Aldrich (St. Louis, MO, USA), Aladdin Bio-Chem Technology (Shanghai, China), and J&K Chemical Technology (Beijing, China).

Derivatization reagent 1-[Bis (dimethylamino) methylene]-1H-1, 2, 3-triazolo[4, 5-b] pyridinium 3oxid hexafluorophosphate (HATU) and 5-dimethylamino-naphthalene-1-sulfonyl chloride (Dns-Cl) were purchased from J&K Chemical Technology (Beijing, China). 5-Dimethylamino-naphthalene-1-sulfonyl piperazine (Dns-PP) was synthesized in our laboratory, as reported previously.[1] Azoxymethane was obtained from Sigma-Aldrich (MO, USA). 5-(bis(methyl-d_3)amino)naphthalene-1-sulfonyl chloride (d_6-Dns-Cl) and N, N-bis(methyl-d_3)-5-(piperazin-1-ylsulfonyl)naphthalen-1-amine (d_6-Dns-PP) were obtained from Wuxi Beita Pharmatech (Jiangsu, China). AOM (azoxymethane) was purchased from Sigma-Aldrich (St. Louis, MO, USA). DSS (dextran sulfate sodium salt, MW 36000 - 50000 kDa) was purchased from MP Biomedicals (Santa Ana, CA, USA).

HPLC-grade acetonitrile and methanol were obtained from Merck (Darmstadt, Germany). Analytical grade sodium carbonate (Na_2CO_3), sodium bicarbonate ($NaHCO_3$), and formic acid were purchased from Nanjing Chemical Reagent (Jiangsu, China). Ultrapure water was prepared by the Milli-Q purification system (Millipore, Watford, UK).

Reagents involved in the chemical derivatization, including Dns-Cl, d_6-Dns-Cl, Dns-PP, d_6-Dns-PP, and HATU, were prepared in acetonitrile. NaOH and formic acid were dissolved in water at 100 mM and 425 mM, respectively. All the solutions were all prepared freshly.

Chemical Derivatization

For amino and phenol submetabolome derivatization, 20 μL of acetonitrile-H_2O (1:1, v/v), 100 μL of carbonate buffer (pH = 10), and 100 μL of Dns-Cl solution (20 mM) were sequentially pipetted into each dried sample. The resulting mixture was incubated at 35°C for 15 minutes. Then, 40 μL of NaOH solution (100 mM) was added to the sample and incubated at 35°C for 10 minutes to quench the reaction. Afterward, 40 μL of formic acid (425 mM) was added to remove excessive alkalinity. For carboxyl submetabolome derivatization, dried samples were in sequence mixed with 20 μL acetonitrile-methanol-H_2O (2:2:1, v/v/v), 40 μL methanol, 20 μL HATU solution (6 mM), and 20 μL Dns-PP solution (12 mM). After blending, the derivatized reaction solution was incubated at 55°C for 20 min.

Our previous study used paired derivatization reagents to provide one-to-one isotope derivatization-insertion standards (ID-ISs). [1-3] ID-ISs were prepared similarly to the actual samples, except that the biological samples were replaced with standard mixtures, and isotope derivatization reagents (i.e., d_6-Dns-Cl and d_6-Dns-PP) were used. ID-ISs and actual samples

were prepared simultaneously. At the end of the reaction, 4 μL of ID-ISs were added to the labeled samples. After vortex mixing and centrifugation at 14,000 rpm for 10 minutes, the supernatant was used for LC-MS/MS analysis.

LC-MS/MS Condition

Targeted metabolite profiling was performed using a liquid chromatography-mass spectrometry/mass spectrometry (LC-MS/MS) approach. Chromatographic separation was achieved on an Agilent Zorbax Eclipse XDB-C18 column (2.1 × 100 mm, 1.8 μm) using a Shimadzu Nexera UPLC system interfaced with an 8060 triple quadruple mass spectrometer (Shimadzu Co., Kyoto, Japan). The mobile phases employed were 0.1% formic acid in water (A) and methanol (B), and the gradient program was as follows: 0-2 min, 30% B; 2-5 min, 30%-52% B; 5-15 min, 52%-65% B; 15-20 min, 65%-78% B; 20-22.5 min, 78% B; 22.5-29 min, 78%-90% B; 29-38 min, 90%-100% B; and 38-40 min, 100%-30% B. The column was maintained at 50°C, and the samples were kept in the autosampler at 4°C. The flow rate was 0.4 mL/min, and injection volumes were 10 μL for the Dns-Cl derivatization method and 5 μL for the Dns-PP derivatization method. For the MS part, the ion source was an electrospray ionization (ESI) source operated in the positive mode. The optimal instrument parameters were set as follows: a spray voltage of 4.5 kV, a nebulizer gas of 3 L/min, a drying gas of 15 L/min, a heat block temperature of 400°C, and a desorption line temperature of 250°C.

References

[1] R. Jiang, Y. Jiao, P. Zhang, Y. Liu, X. Wang, Y. Huang, Z. Zhang, F. Xu, Anal Chem. 2017, 89, 12223.

[2] W. Li, P. Zhang, X. Hou, T. Tang, S. Li, R. Sun, Z. Zhang, F. Xu, Anal Chim Acta. 2022, 1193, 339399.

[3] S. Qin, M. Gao, Q. Zhang, Q. Xiao, J. Fu, Y. Tian, Y. Jiao, Z. Zhang, P. Zhang, F. Xu, Anal Chem. 2023, 95, 10034.

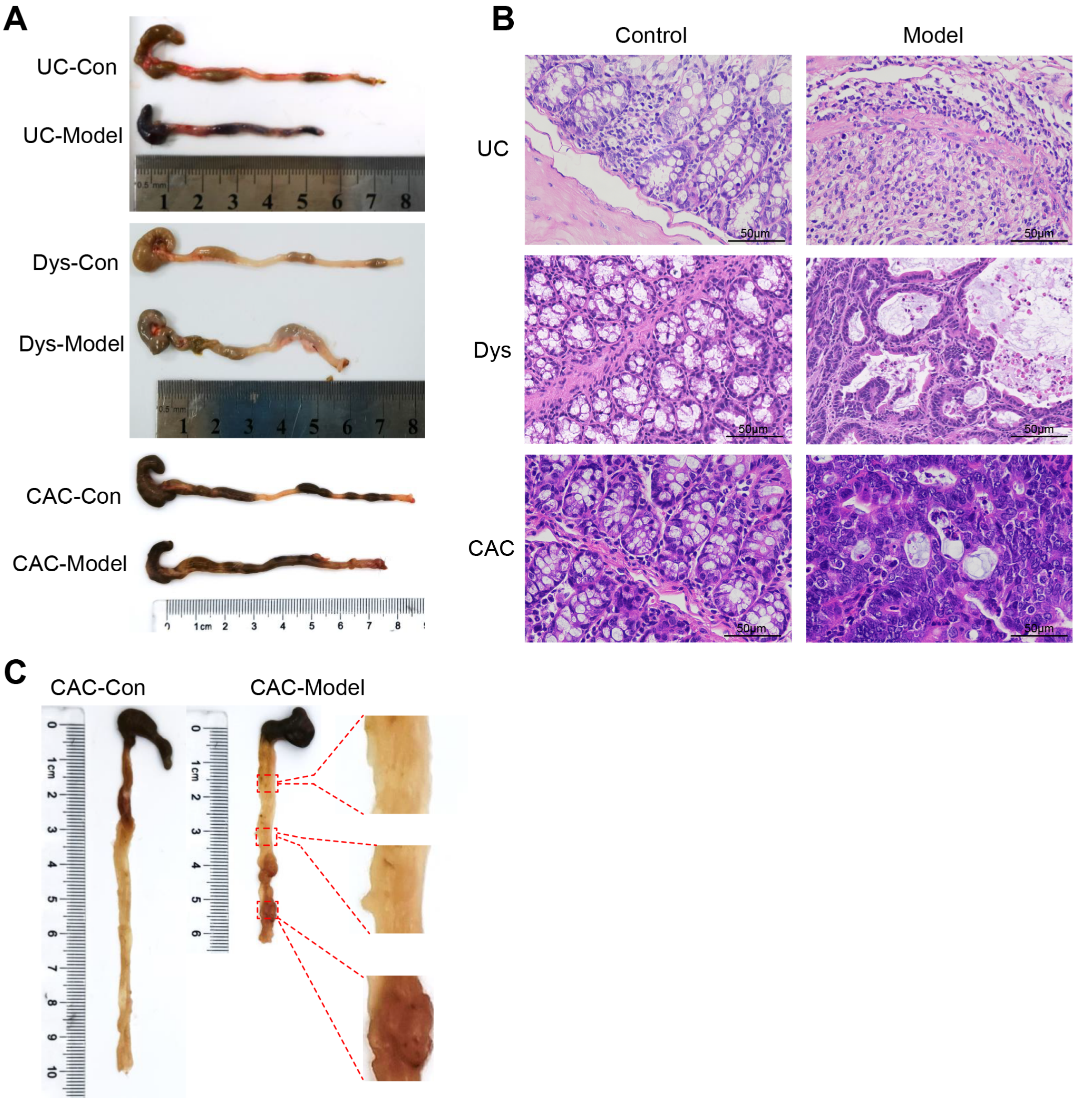

Figure S1. Establishment of the UC-CRC mouse model. A) Colorectal length comparison between control mice and mice in the UC, Dys, and CAC groups. B) H&E staining of colorectal tissue from control mice and mice at various stages of the model (UC, Dys, CAC). C) Cross-sectional view of the colorectum from control mice and CAC group mice, showing tumor development in the colorectum of AOM-DSS treated mice.

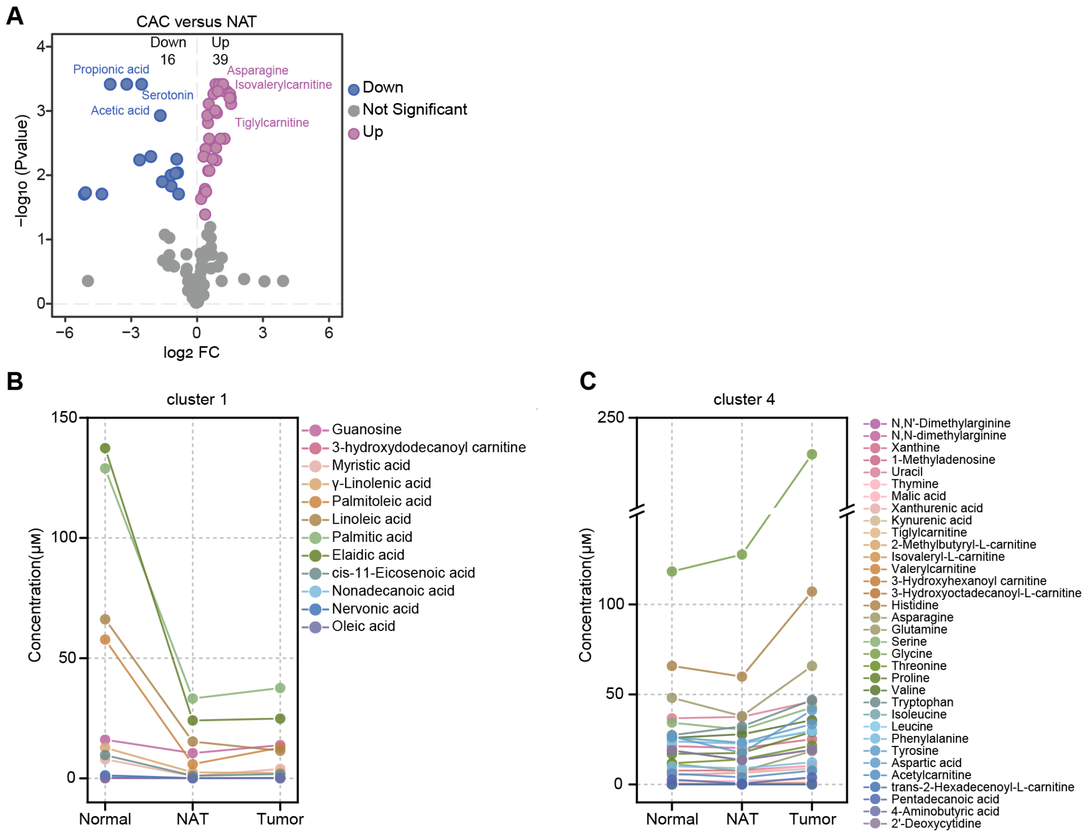

Figure S2. Spatially differentiated metabolites and cluster analysis. A) Volcano plot displaying differentially expressed metabolites between tumor tissue and NAT in the CAC stage. Significant changes were defined using the criteria of FC (Fold change) ≥ 1.5 or FC ≤ 0.667, and $P < 0.05$. Metabolites significantly increased or decreased are indicated in pink and blue, respectively. B) Dynamics of down-regulated metabolites in cluster 1, with dots representing the mean relative abundance values. C) Dynamics of up-regulated metabolites in cluster 4, with dots representing the mean relative abundance values.

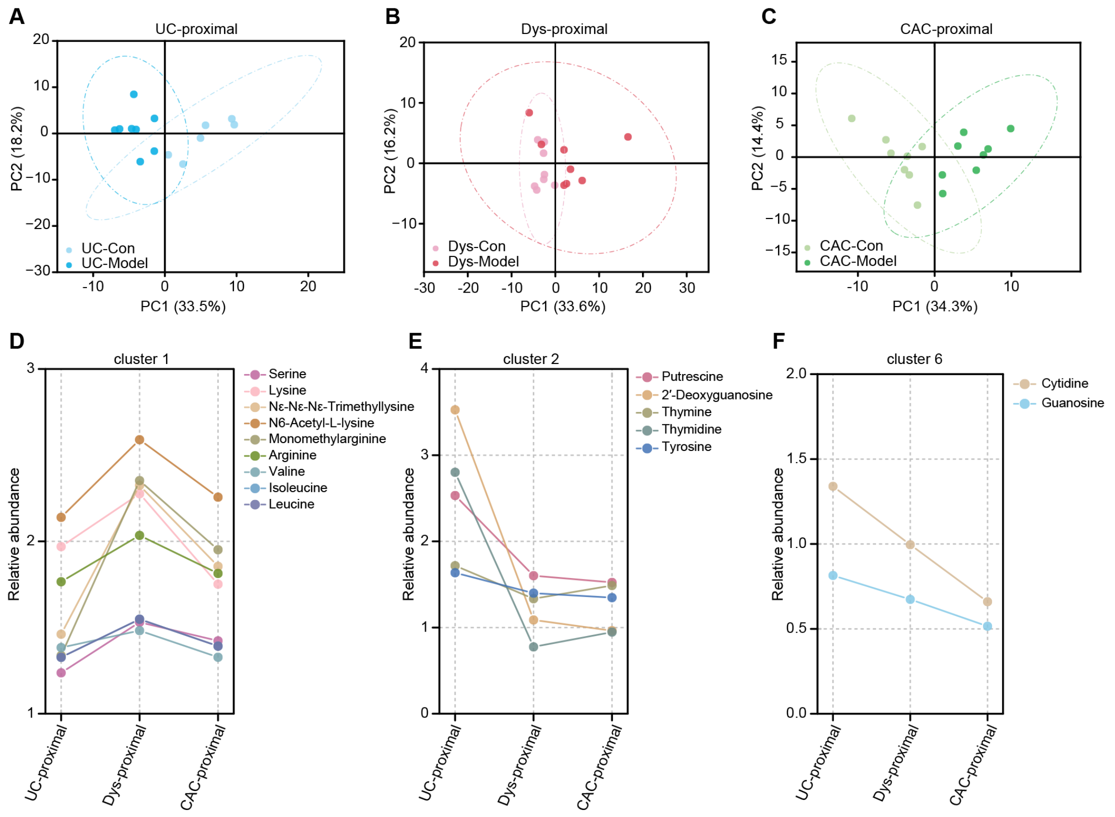

Figure S3. Temporal metabolic changes from UC to CAC in the proximal colon. A-C) PCA score plots illustrating the metabolomics data of the proximal colon at the UC, Dys, and CAC stages. Light blue indicates UC stage control group, blue indicates UC stage model group, light pink indicates Dys stage control group, pink indicates Dys stage model group, light green indicates CAC stage control group, green indicates CAC stage model group. D-E) Dynamics of up-regulated metabolites in clusters 1 and 2, with dots representing mean relative abundance values. F) Dynamics of down-regulated metabolites in cluster 5, with dots representing mean relative abundance values.

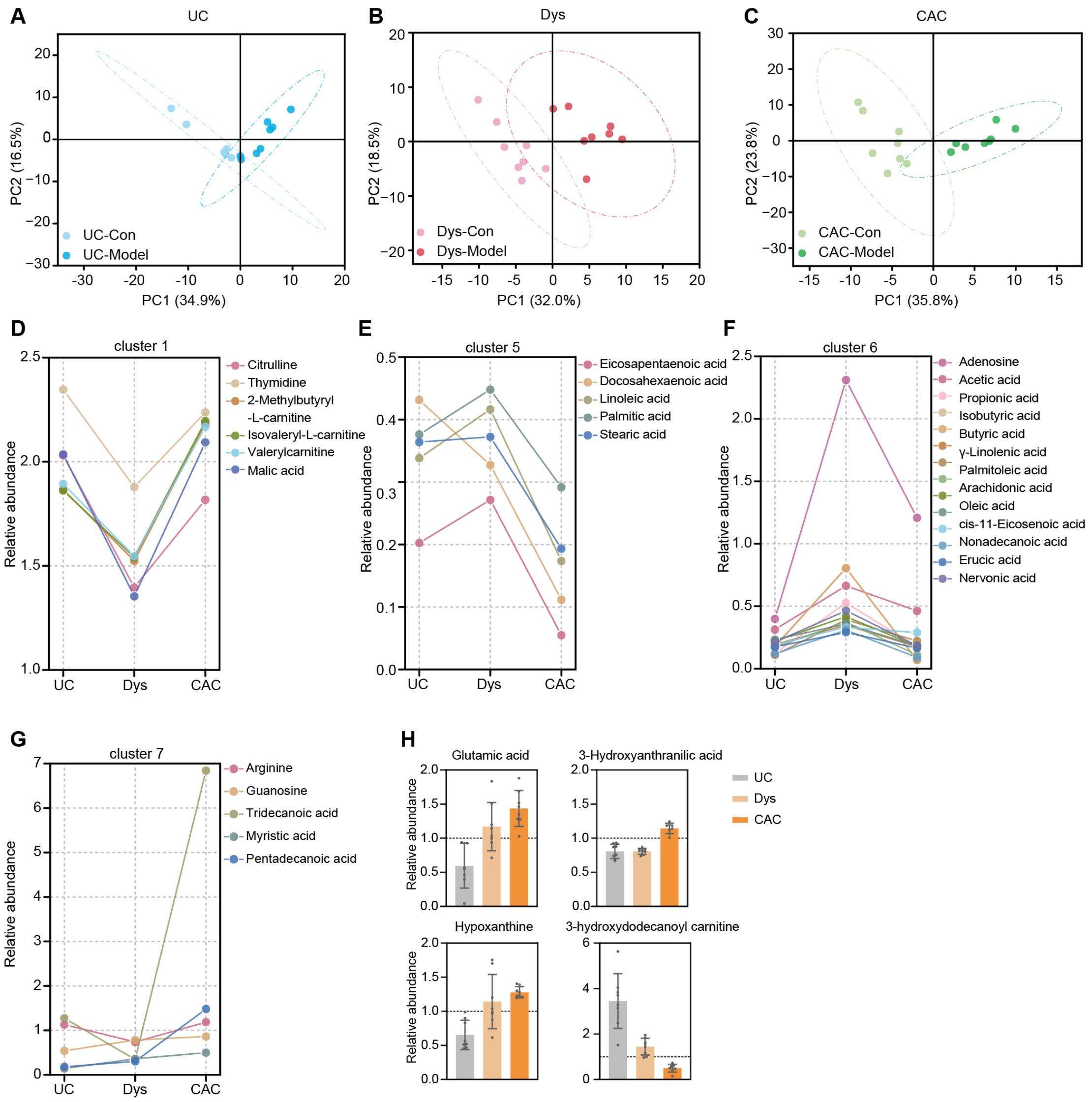

Figure S4. Temporal metabolic changes from UC to CAC in the distal colon. A-C) PCA score plots showing metabolomics data of the distal colon at the UC, Dys, and CAC stages. D) Dynamics of up-regulated metabolites in cluster 1, with dots representing mean relative abundance values. E-G) Dynamics of down-regulated metabolites in clusters 5, 6, and 7, with dots representing mean relative abundance values. H) Opposing trends in metabolite changes at different stages in the distal colon.

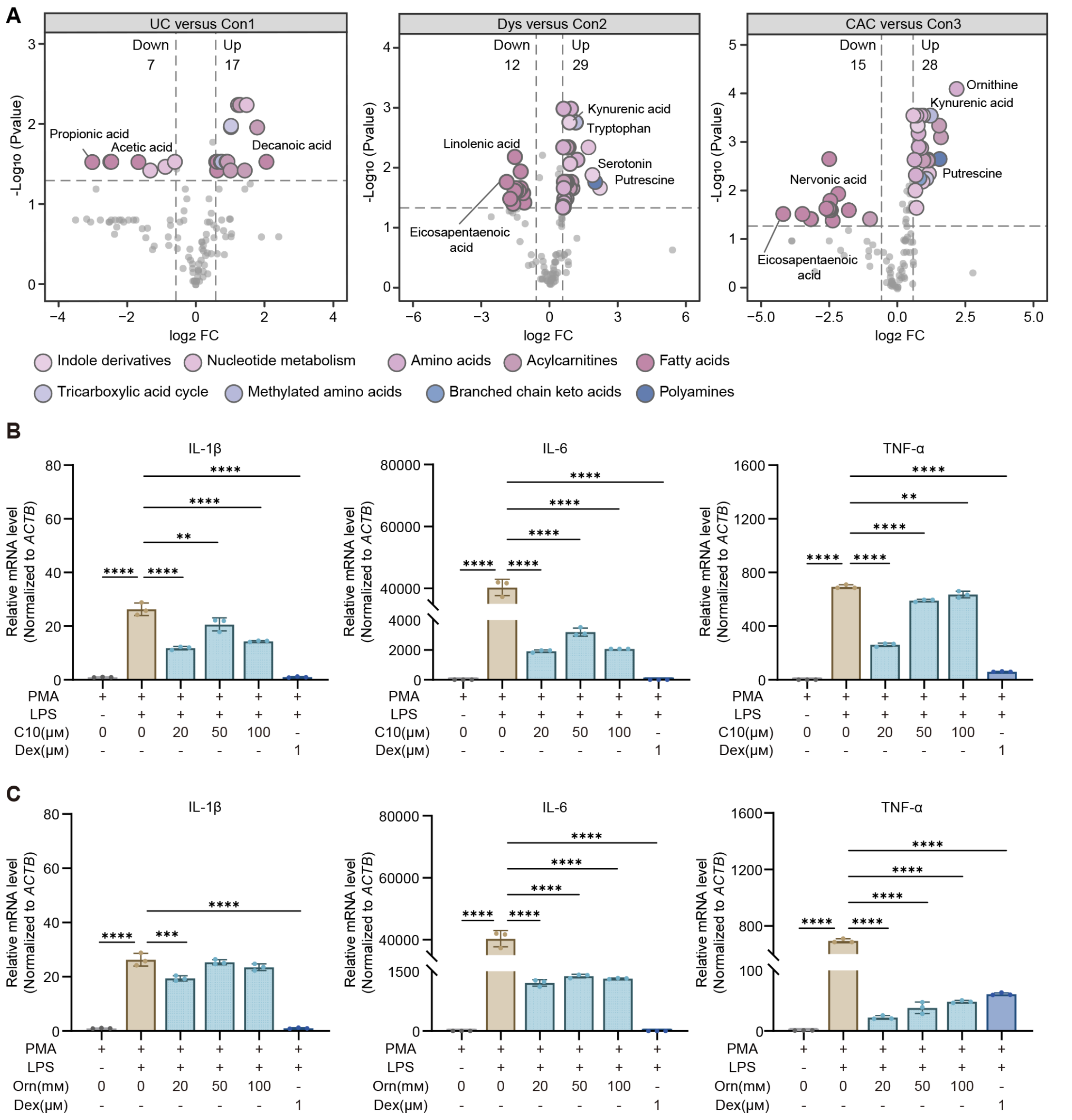

Figure S5. Investigation on the function of key metabolites including C10, Orn. A) Volcano plots showing differential metabolites in the distal colon between the model group and the paired control group at the UC, Dys, and CAC stages. Significant changes were defined using the criteria of FC ≥ 1.5 or FC ≤ 0.667, and $P < 0.05$. Metabolites significantly increased or decreased are indicated in pink and blue, respectively. B-C) RT-qPCR analysis of TNF-α, IL-1β, and IL-6 in LPS-induced THP-1 macrophages treated with C10 (B), Orn (C), and Dex (n = 3). All data are expressed as means ± SD. One-way ANOVA with Bonferroni's correction was applied in (B-C). *$P < 0.05$, **$P < 0.01$, ***$P < 0.001$, ****$P < 0.0001$.

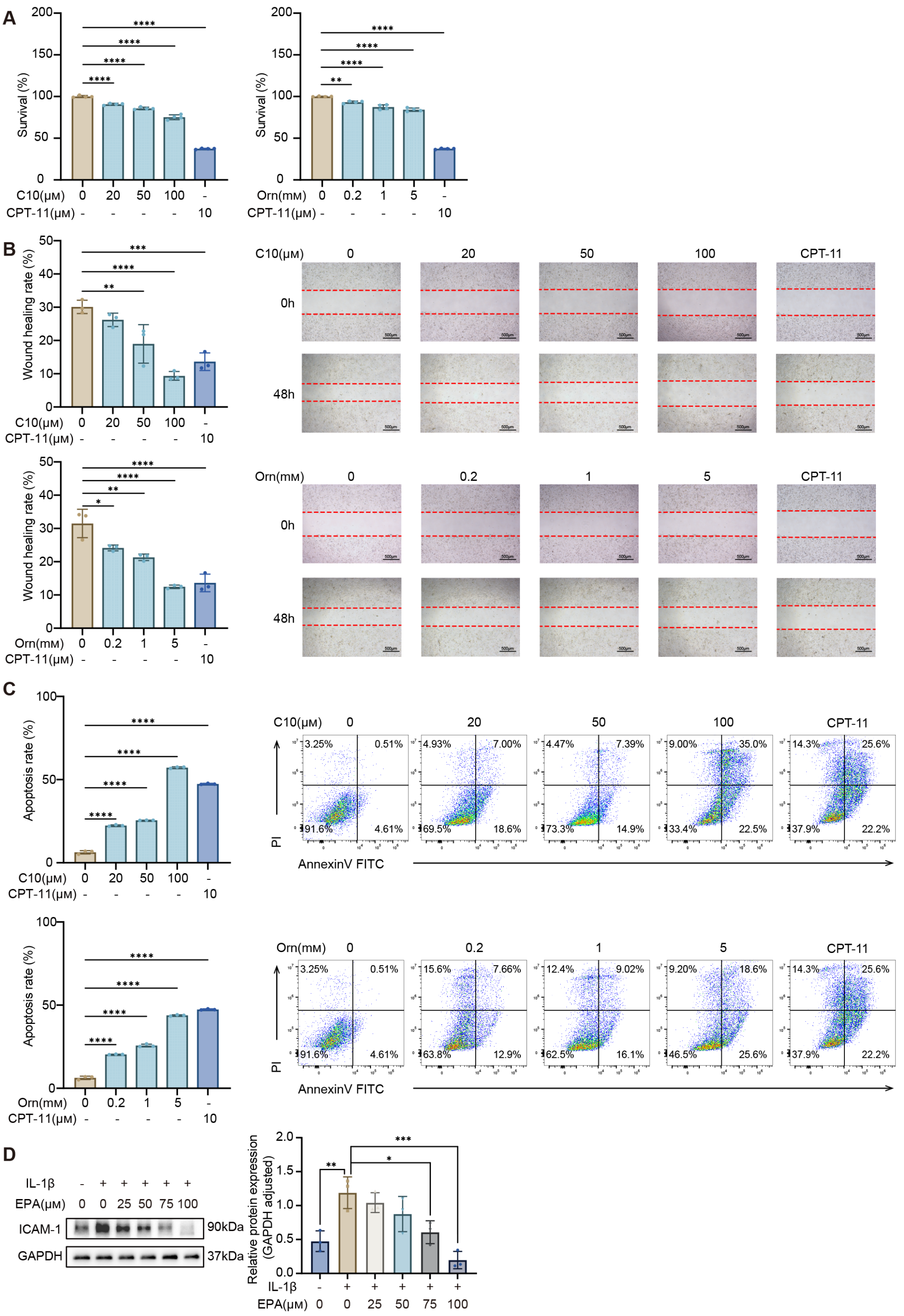

Figure S6. Investigation on the function of key metabolites including C10, Orn, and EPA. A) Cell viability of HCT116 cells treated with Orn, C10 and CPT-11 (n = 3). B) Scratch assay of HCT116 cells treated with Orn, C10 and CPT-11 after 0 and 48h of incubation (n = 3). C) The effects of Orn, C10 and CPT-11 on apoptosis of HCT116 cells (n = 3). D) Western blot analysis of ICAM-1 expression in CCD841 CoN cells induced by IL-1β and treated with EPA (n = 3). All data are expressed as means ± SD. One-way ANOVA with Bonferroni's correction was applied in (A-D). *$P < 0.05$, **$P < 0.01$, ***$P < 0.001$, ****$P < 0.0001$.

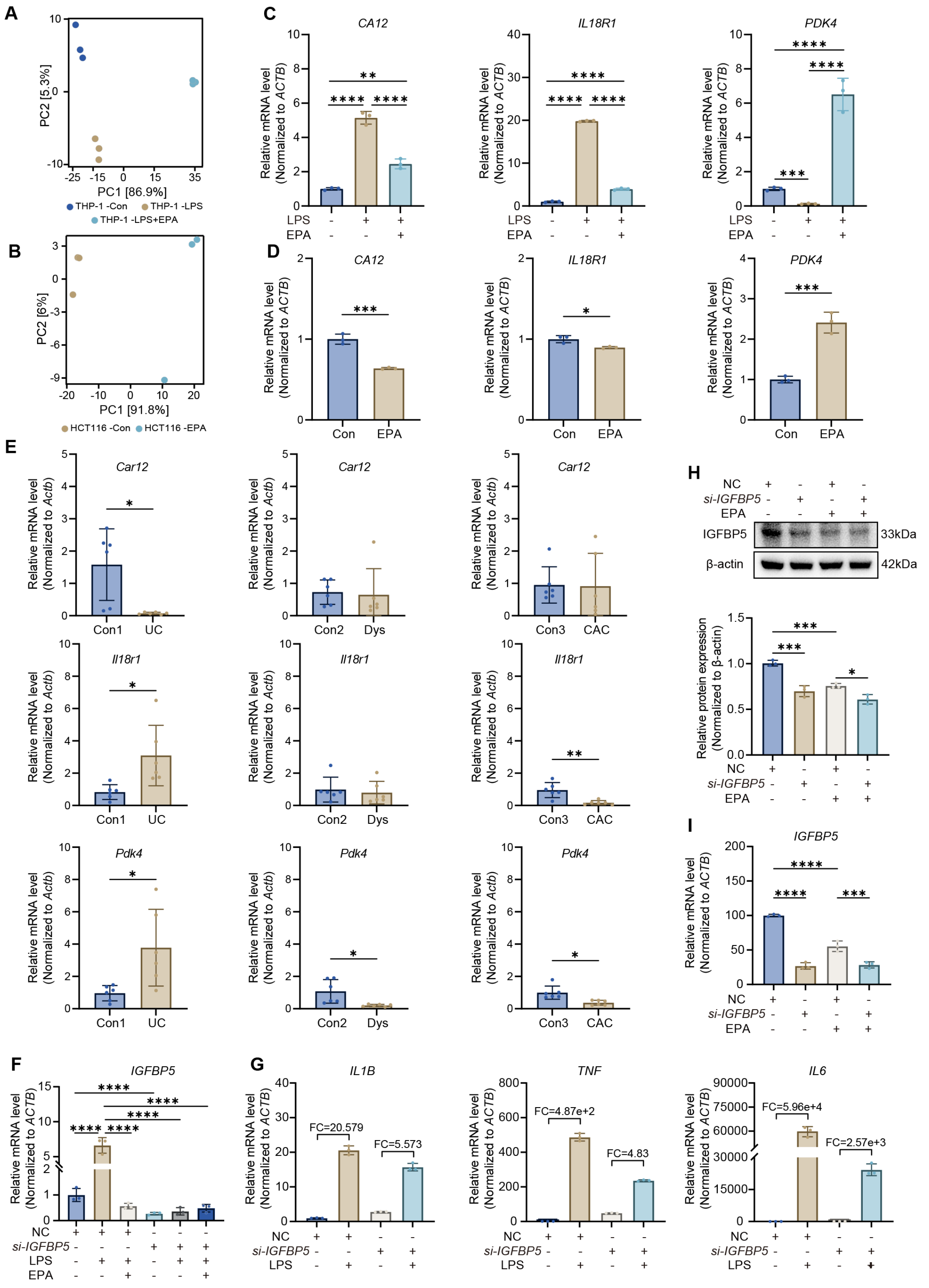

Figure S7. Screening and validation of IGFBP5. A) PCA plot of RNA-seq data in THP-1 macrophages. Blue represents the control group, yellow for LPS-treated group, and pale blue for LPS+EPA-treated group. B) PCA plot of RNA-seq data in HCT116 cells. Yellow represents the control group, and pale blue for EPA-treated group. C) RT-qPCR

analysis of *CA12*, *IL18R1*, and *PDK4* in LPS-induced THP-1 macrophages treated with EPA. D) RT-qPCR analysis of *CA12*, *IL18R1*, and *PDK4* in HCT116 cells treated with EPA. E) RT-qPCR analysis of *Car12*, *Il18r1*, and *Pdk4* in the distal colon animal at different stages. F) RT-qPCR analysis of *IGFBP5* expression after knockdown of *IGFBP5* in LPS-induced THP-1 macrophages. G) RT-qPCR analysis after knockdown of *IGFBP5* on inflammatory genes *TNF*, *IL6*, and *IL1B* in LPS-induced THP-1 macrophages. H) Western blot analysis of IGFBP5 protein expression in HCT116 cells with *IGFBP5* knockdown. I) RT-qPCR analysis of *IGFBP5* expression after EPA treatment in HCT116 cells with *IGFBP5* knockdown. All data are presented as means ± SD. Statistical significance was determined using an unpaired two-tailed Student's t-test (D-E) or one-way ANOVA with Bonferroni's correction (C, F-I). *$P < 0.05$, **$P < 0.01$, ***$P < 0.001$, ****$P < 0.0001$.

Table S1. List of standards and labeled analytes

No	Metabolites	CAS No	Class	Reaction reagent	Molecular weight	Parent ion (m/z)	CE (V)	Fragment (m/z)	Retention time (min)
1	L-Alanine	56-41-7	Amino acids	Dns-Cl	89.09	323.2	-29	157.15	6.93
2	L-Arginine	74-79-3	Amino acids	Dns-Cl	174.2	408.1	-36	170.2	3.06
3	L-Asparagine	70-47-3	Amino acids	Dns-Cl	132.12	366.1	-35	170.1	3.95
4	L-Aspartic acid	56-84-8	Amino acids	Dns-PP	133.1	736.95	-35	320.1	12.789
5	L-Citrulline	372-75-8	Amino acids	Dns-Cl	175.19	409.45	-35	170.1	4.91
6	L-Glutamine	56-85-9	Amino acids	Dns-Cl	146.14	380.2	-27	170.2	4.198
7	L-Glutamic acid	56-86-0	Amino acids	Dns-PP	147.13	449.55	-35	170	6.576
8	L-Glycine	56-40-6	Amino acids	Dns-Cl	75.07	309	-29	157.1	5.406
9	L-Histidine	71-00-1	Amino acids	Dns-Cl	155.15	622.7	-35	170	17.903
10	L-Isoleucine	73-32-5	Amino acids	Dns-Cl	131.17	365	-30	157.25	11.43
11	L-Leucine	61-90-5	Amino acids	Dns-Cl	131.17	365	-30	157.25	11.645
12	L-Lysine	56-87-1	Amino acids	Dns-Cl	146.19	613.2	-30	170.1	16.01
13	Ornithine	70-26-8	Amino acids	Dns-Cl	132.16	599.2	-44	170	15.21
14	L-Phenylalanine	63-91-2	Amino acids	Dns-Cl	165.19	399.1	-25	170.2	11.744
15	L-Proline	147-85-3	Amino acids	Dns-Cl	115.13	349	-23	170.25	8.44
16	L-Serine	56-45-1	Amino acids	Dns-Cl	105.09	339	-21	170.1	5.1437
17	L-Threonine	72-19-5	Amino acids	Dns-Cl	119.12	353.2	-35	157.2	5.791
18	L-Tryptophan	73-22-3	Amino acids	Dns-Cl	204.23	438.2	-29	170.1	9.92

No	Metabolites	CAS No	Class	Reaction reagent	Molecular weight	Parent ion (m/z)	CE (V)	Fragment (m/z)	Retention time (min)
19	L-Tyrosine	60-18-4	Amino acids	Dns-Cl	181.19	648.2	-42	170.1	20.451
20	L-Valine	72-18-4	Amino acids	Dns-Cl	117.15	351.1	-30	157.15	9.42
21	Ketoleucine	816-66-0	branched chain keto acids	Dns-PP	130.1418	432.25	-26	170.2	16.18
22	α-Ketoisovaleric acid	759-05-7	branched chain keto acids	Dns-PP	116.12	418.25	-24	170.2	12.731
23	3-Methyl-2-oxovaleric acid	1460-34-0	branched chain keto acids	Dns-PP	130.14	432.25	-24	170	15.753
24	L-Carnitine	541-15-1	Acylcarnitines	Dns-PP	161.2	463.6	-25	386.15	4.99
25	trans-2-Dodecenoyl-L-carnitine	1631992-06-7	Acylcarnitines	Dns-PP	341.49	643.91	-30	386.15	19.792
26	3-hydroxydodecanoyl carnitine	1469900-99-9	Acylcarnitines	Dns-PP	359.5	661.92	-33	386.15	18.755
27	Myristoyl-L-carnitine	25597-07-3	Acylcarnitines	Dns-PP	371.55	673.97	-30	386.15	22.02
28	trans-2-Tetradecenoyl-L-carnitine	1256380-19-4	Acylcarnitines	Dns-PP	369.54	671.96	-31	386.15	21.574
29	Palmitoyl-L-carnitine	2364-67-2	Acylcarnitines	Dns-PP	399.61	702.03	-32	386.15	24.067
30	trans-2-Hexadecenoyl-L-carnitine	26145-55-1	Acylcarnitines	Dns-PP	397.59	700.01	-34	386.15	23.445
31	Stearoyl-L-carnitine	25597-09-5	Acylcarnitines	Dns-PP	427.66	730.08	-34	386.15	24.585
32	Oleoyl-L-carnitine	38677-66-6	Acylcarnitines	Dns-PP	425.64	728.06	-33	386.15	24.281
33	cis,cis-9,12-Octadecadienoyl-L-carnitine	36816-10-1	Acylcarnitines	Dns-PP	423.63	726.05	-33	386.15	23.433

No	Metabolites	CAS No	Class	Reaction reagent	Molecular weight	Parent ion (m/z)	CE (V)	Fragment (m/z)	Retention time (min)
34	3-Hydroxyoctadecanoyl-L-carnitine	1469901-05-0	Acylcarnitines	Dns-PP	443.66	746.08	-37	386.15	24.272
35	L-Acetylcarnitine	3040-38-8	Acylcarnitines	Dns-PP	203.24	505.25	-27	386.15	5.43
36	Butyrylcarnitine	25576-40-3	Acylcarnitines	Dns-PP	231.29	533.71	-26	386.15	7.277
37	2-Methylbutyryl-L-carnitine	256928-75-3	Acylcarnitines	Dns-PP	245.32	547.74	-27	386.15	8.007
38	3-Hydroxybutyryl-carnitine	875668-57-8	Acylcarnitines	Dns-PP	247.288	549.7	-28	386.15	5.72
39	Valerylcarnitine	40225-14-7	Acylcarnitines	Dns-PP	245.32	547.74	-27	386.15	8.328
40	Tiglylcarnitine	64681-36-3	Acylcarnitines	Dns-PP	243.3	545.1	-25	386.15	6.96
41	Hexanoylcarnitine	22671-29-0	Acylcarnitines	Dns-PP	259.34	561.15	-27	386.15	9.33
42	3-Hydroxyhexanoyl carnitine	1469900-93-3	Acylcarnitines	Dns-PP	275.34	577.76	-27	386.15	7.823
43	L-Octanoylcarnitine	25243-95-2	Acylcarnitines	Dns-PP	287.4	588.9	-29	386.15	13.45
44	Isobutyryl-L-carnitine	25518-49-4	Acylcarnitines	Dns-PP	231.29	533.7	-26	386.15	7.164
45	Isovaleryl-L-carnitine	31023-24-2	Acylcarnitines	Dns-PP	245.32	547.74	-27	386.15	8.214
46	Decanoic acid	334-48-5	Fatty acids	Dns-PP	172.26	474.1	-23	320.1	24.385
47	Tridecanoic acid	638-53-9	Fatty acids	Dns-PP	214.34	516.4	-23	320.1	26.405
48	Myristic acid	544-63-8	Fatty acids	Dns-PP	228.37	530.4	-23	320.1	27.252
49	Pentadecanoic acid	1002-84-2	Fatty acids	Dns-PP	242.4	544.2	-25	320.1	28.193
50	Palmitic acid	57-10-3	Fatty acids	Dns-PP	256.42	558.2	-25	320.1	29.492
51	Palmitoleic acid	373-49-9	Fatty acids	Dns-PP	254.41	556.4	-23	320.1	27.766

No	Metabolites	CAS No	Class	Reaction reagent	Molecular weight	Parent ion (m/z)	CE (V)	Fragment (m/z)	Retention time (min)
52	Stearic acid	57-11-4	Fatty acids	Dns-PP	284.48	586.3	-26	320.1	31.537
53	elaidic acid	112-79-8	Fatty acids	Dns-PP	282.46	584.88	-27	320.1	30.558
54	Oleic acid	112-80-1	Fatty acids	Dns-PP	282.46	584.3	-27	320.1	30.297
55	Linoleic acid	60-33-3	Fatty acids	Dns-PP	280.45	582.3	-24	320.1	28.563
56	γ-Linolenic acid	506-26-3	Fatty acids	Dns-PP	278.43	580.2	-24	320.1	27.456
57	Nonadecanoic acid	646-30-0	Fatty acids	Dns-PP	298.5	600.5	-26	320.1	32.128
58	Acetic acid	64-19-7	Fatty acids	Dns-PP	60.05	362.2	-18	320.1	8.17
59	cis-11-Eicosenoic acid	5561-99-9	Fatty acids	Dns-PP	310.51	612.3	-27	320.1	31.78
60	Arachidonic acid	506-32-1	Fatty acids	Dns-PP	304.47	606.2	-24	320.1	28.491
61	cis-5,8,11,14,17-Eicosapentaenoic acid	10417-94-4	Fatty acids	Dns-PP	302.45	604.2	-23	320.1	27.474
62	Behenic acid	112-85-6	Fatty acids	Dns-PP	340.58	642.5	-29	320.1	33.975
63	Erucic acid	112-86-7	Fatty acids	Dns-PP	338.57	640.5	-27	320.1	32.914
64	cis-4,7,10,13,16,19-Docosahexaenoic acid	6217-54-5	Fatty acids	Dns-PP	328.49	630.3	-21	320.1	28.312
65	Nervonic acid	506-37-6	Fatty acids	Dns-PP	366.62	668.6	-29	320.1	33.977
66	Propionic acid	79-09-4	Fatty acids	Dns-PP	74.08	376.2	-18	320.1	9.634
67	Butyric acid	107-92-6	Fatty acids	Dns-PP	88.11	390.3	-20	320.1	11.71
68	Isobutyric acid	79-31-2	Fatty acids	Dns-PP	88.11	390.3	-20	320.1	11.38

No	Metabolites	CAS No	Class	Reaction reagent	Molecular weight	Parent ion (m/z)	CE (V)	Fragment (m/z)	Retention time (min)
69	2-Methyl butyric acid	116-53-0	Fatty acids	Dns-PP	102.13	404.1	-19	320.1	13.533
70	Isovaleric acid	503-74-2	Fatty acids	Dns-PP	102.13	404.1	-19	320.1	13.9
71	Valeric acid	109-52-4	Fatty acids	Dns-PP	102.13	404.1	-19	320.1	14.425
72	Nonanoic acid	112-05-0	Fatty acids	Dns-PP	158.24	460.3	-22	320.1	22.985
73	4-Methyloctanoic acid	54947-74-9	Fatty acids	Dns-PP	158.24	460.3	-22	320.1	22.528
74	2-Oxoadipic acid	3184-35-8	Indole derivatives	Dns-PP	160.12	763.95	-40	170	19.675
75	Serotonin	50-67-9	Indole derivatives	Dns-Cl	176.22	643.2	-47	170.05	21.057
76	5-Methoxyindoleacetic acid	3471-31-6	Indole derivatives	Dns-PP	205.22	507	-23	320.1	11.978
77	6-Hydroxymelatonin	2208-41-5	Indole derivatives	Dns-Cl	248.28	482.2	-33	170.05	11.893
78	3-Hydroxyanthranilic acid	548-93-6	Indole derivatives	Dns-Cl	153.14	387.2	-19	170.1	15.55
79	5-Hydroxyindoleacetate	54-16-0	Indole derivatives	Dns-Cl	191.18	425.1	-27	170.1	11.99
80	Indole-3-carboxaldehyde	487-89-8	Indole derivatives	Dns-Cl	145.16	379.15	-22	170.1	18.827
81	DL-Indole-3-lactic acid	832-97-3	Indole derivatives	Dns-PP	205.21	507.15	-30	169.9	11.956
82	Kynurenic acid	492-27-3	Indole derivatives	Dns-Cl	189.17	423.15	-28	170.15	17.17
83	Kynurenine	2922-83-0	Indole derivatives	Dns-Cl	208.21	442.2	-33	170.15	9.255
84	2-Picolinic acid	98-98-6	Indole derivatives	Dns-PP	123.11	425.05	-29	170.1	9.796
85	Xanthurenic acid	59-00-7	Indole derivatives	Dns-Cl	205.17	439.1	-25	170.1	10.21
86	NG-NG'-Dimethyl-L-arginine	30344-00-4	Methylated amino acids	Dns-Cl	202.25	435.8	-35	170.15	4.49
87	NG,NG-Dimethy-L-arginine	30315-93-6	Methylated amino acids	Dns-Cl	188.23	421.9	-34	170.05	12.586

No	Metabolites	CAS No	Class	Reaction reagent	Molecular weight	Parent ion (m/z)	CE (V)	Fragment (m/z)	Retention time (min)
88	Nε-Acetyl-L-lysine	692-04-6	Methylated amino acids	Dns-Cl	188.22	422.15	-22	170.15	6.54
89	NG-Methyl-L-arginine	17035-90-4	Methylated amino acids	Dns-Cl	188.23	422	-34	170.15	4.12
90	Adenine	73-24-5	nucleotide metabolism	Dns-Cl	135.13	368.9	-24	170	9.572
91	Adenosine	58-61-7	nucleotide metabolism	Dns-Cl	267.24	501.05	-22	136	8.094
92	Cytidine	65-46-3	nucleotide metabolism	Dns-Cl	243.22	477.1	-20	112.1	5.746
93	2'-Deoxycytidine	951-77-9	nucleotide metabolism	Dns-Cl	227.22	461.1	-20	112.05	6.753
94	2'-Deoxyguanosine	312693-72-4	nucleotide metabolism	Dns-Cl	267.24	501.5	-20	152.2	7.629
95	Guanosine	118-00-3	nucleotide metabolism	Dns-Cl	283.24	517	-35	135	6.727
96	Hypoxanthine	68-94-0	nucleotide metabolism	Dns-Cl	136.11	370.4	-35	170	7.95
97	1-Methyladenine	5142-22-3	nucleotide metabolism	Dns-Cl	149.15	382.9	-28	170.1	5.849
98	1-Methyladenosine	15763-06-1	nucleotide metabolism	Dns-Cl	281.27	515	-32	150.1	6.971
99	5-Methylcytidine	2140-61-6	nucleotide metabolism	Dns-Cl	257.24	491.1	-19	126.2	5.951
100	Thymidine	50-89-5	nucleotide metabolism	Dns-Cl	242.23	476.5	-35	127	8.667
101	Thymine	65-71-4	nucleotide metabolism	Dns-Cl	126.12	360.4	-35	170/157	9.79
102	Uracil	66-22-8	nucleotide metabolism	Dns-Cl	112.09	346.35	-35	157	7.96
103	Uridine	58-96-8	nucleotide metabolism	Dns-Cl	244.2	478.45	-35	113	6.553
104	Xanthine	69-89-6	nucleotide metabolism	Dns-Cl	152.11	387.4	-50	170	7.362
105	3-Hydroxybutyric acid	625-72-9	Others	Dns-PP	104.1	406.52	-20	320.1	8.482
106	Ethanolamine	141-43-5	Others	Dns-Cl	61.08	295	-35	157	5.849

No	Metabolites	CAS No	Class	Reaction reagent	Molecular weight	Parent ion (m/z)	CE (V)	Fragment (m/z)	Retention time (min)
107	4-Aminobutyric acid	56-12-2	Others	Dns-CL	103.12	337	-20	169.5	6.52
108	Putrescine	110-60-1	Polyamines	Dns-Cl	88.15	555.2	-37	170.2	17.536
109	Citric acid	77-92-9	tricarboxylic acid cycle	Dns-PP	192.12	1096.9	-53	320.2	25.139
110	Fumaric acid	110-17-8	tricarboxylic acid cycle	Dns-PP	116.07	719.1	-51	170.2	18.914
111	isocitric acid	1637-73-6	tricarboxylic acid cycle	Dns-PP	192.123	1097.4	-55	170	24.707
112	α-Ketoglutaric acid	328-50-7	tricarboxylic acid cycle	Dns-PP	146.1	749.95	-35	320.1	20.581
113	Malic acid	97-67-6	tricarboxylic acid cycle	Dns-PP	134.09	737.4	-59	170.2	17.92
114	Pyruvic acid	127-17-3	tricarboxylic acid cycle	Dns-PP	88.06	390	-25	170.2	8.669
115	Succinic acid	110-15-6	tricarboxylic acid cycle	Dns-PP	118.09	721.45	-60	170.2	18.562

Table S2. Clinicopathological characteristics of the included samples in GSE16879 dataset

Source	Tissue Location	Diagnosis	inflammation	Method
GSM364627	colon	control individuals	normal area	endoscopy biopsy
GSM364628	colon	control individuals	normal area	endoscopy biopsy
GSM364629	colon	control individuals	normal area	endoscopy biopsy
GSM364630	colon	control individuals	normal area	endoscopy biopsy
GSM364631	colon	control individuals	normal area	endoscopy biopsy
GSM364632	colon	control individuals	normal area	endoscopy biopsy
GSM364633	colon	Ulcerative colitis	Inflamed area	endoscopy biopsy
GSM364634	colon	Ulcerative colitis	Inflamed area	endoscopy biopsy
GSM364635	colon	Ulcerative colitis	Inflamed area	endoscopy biopsy
GSM364636	colon	Ulcerative colitis	Inflamed area	endoscopy biopsy
GSM364637	colon	Ulcerative colitis	Inflamed area	endoscopy biopsy
GSM364638	colon	Ulcerative colitis	Inflamed area	endoscopy biopsy
GSM364639	colon	Ulcerative colitis	Inflamed area	endoscopy biopsy
GSM364640	colon	Ulcerative colitis	Inflamed area	endoscopy biopsy
GSM364641	colon	Ulcerative colitis	Inflamed area	endoscopy biopsy
GSM364642	colon	Ulcerative colitis	Inflamed area	endoscopy biopsy
GSM364643	colon	Ulcerative colitis	Inflamed area	endoscopy biopsy
GSM364644	colon	Ulcerative colitis	Inflamed area	endoscopy biopsy
GSM364645	colon	Ulcerative colitis	Inflamed area	endoscopy biopsy
GSM364646	colon	Ulcerative colitis	Inflamed area	endoscopy biopsy
GSM364647	colon	Ulcerative colitis	Inflamed area	endoscopy biopsy
GSM364648	colon	Ulcerative colitis	Inflamed area	endoscopy biopsy
GSM364649	colon	Ulcerative colitis	Inflamed area	endoscopy biopsy
GSM364650	colon	Ulcerative colitis	Inflamed area	endoscopy biopsy
GSM364651	colon	Ulcerative colitis	Inflamed area	endoscopy biopsy
GSM364652	colon	Ulcerative colitis	Inflamed area	endoscopy biopsy
GSM364653	colon	Ulcerative colitis	Inflamed area	endoscopy biopsy
GSM364654	colon	Ulcerative colitis	Inflamed area	endoscopy biopsy
GSM364655	colon	Ulcerative colitis	Inflamed area	endoscopy biopsy
GSM364656	colon	Ulcerative colitis	Inflamed area	endoscopy biopsy

Table S3. Clinicopathological characteristics of the included samples in GSE4183 dataset

Source	Tissue Location	Diagnose	Method
GSM95473	colon	control individuals	colon biopsy
GSM95474	colon	control individuals	colon biopsy
GSM95475	colon	control individuals	colon biopsy
GSM95476	colon	control individuals	colon biopsy
GSM95477	colon	control individuals	colon biopsy
GSM95478	colon	control individuals	colon biopsy
GSM95479	colon	control individuals	colon biopsy
GSM95480	colon	control individuals	colon biopsy
GSM95496	carcinomas	Colorectal cancer	colon biopsy
GSM95497	carcinomas	Colorectal cancer	colon biopsy
GSM95498	carcinomas	Colorectal cancer	colon biopsy
GSM95499	carcinomas	Colorectal cancer	colon biopsy
GSM95500	carcinomas	Colorectal cancer	colon biopsy
GSM95501	carcinomas	Colorectal cancer	colon biopsy
GSM95502	carcinomas	Colorectal cancer	colon biopsy
GSM95503	carcinomas	Colorectal cancer	colon biopsy
GSM95504	carcinomas	Colorectal cancer	colon biopsy
GSM95505	carcinomas	Colorectal cancer	colon biopsy
GSM95506	carcinomas	Colorectal cancer	colon biopsy
GSM95507	carcinomas	Colorectal cancer	colon biopsy
GSM95508	carcinomas	Colorectal cancer	colon biopsy
GSM95509	carcinomas	Colorectal cancer	colon biopsy
GSM95510	carcinomas	Colorectal cancer	colon biopsy
GSM95473	colon	control individuals	colon biopsy
GSM95474	colon	control individuals	colon biopsy
GSM95475	colon	control individuals	colon biopsy
GSM95476	colon	control individuals	colon biopsy
GSM95477	colon	control individuals	colon biopsy
GSM95478	colon	control individuals	colon biopsy
GSM95479	colon	control individuals	colon biopsy

Table S4. The primers used in RT-qPCR

Oligonucleotide	Primer	Sequences (5′-3′)
Homo *IL6*	Forward primer	GAAAGCAGCAAAGAGGCACT
	Reverse primer	TTTCACCAGGCAAGTCTCCT
Homo *IL1B*	Forward primer	GGCATCCAGCTACGAATCTC
	Reverse primer	GAACCAGCATCTTCCTCAGC
Homo *TNF*	Forward primer	AACCTCCTCTCTGCCATCAA
	Reverse primer	GGAAGACCCCTCCCAGATAG
Homo *IGFBP5*	Forward primer	TGACCGCAAAGGATTCTACAAG
	Reverse primer	CGTCAACGTACTCCATGCCT
Homo *CA12*	Forward primer	AGTGACATCCTCCAGTATGACG
	Reverse primer	GTGGCACTGTAGCGAGACT
Homo *IL18R1*	Forward primer	AAGAACGCCGAGTTTGAAGAT
	Reverse primer	GAGCAGTTGAGCCTTACGTTT
Homo *PDK4*	Forward primer	CCTTTGGCTGGTTTTGGTTA
	Reverse primer	CCTGCTTGGGATACACCAGT
Mus *Car12*	Forward primer	ATAGGCTCCGCCAATCCATC
	Reverse primer	CATAGCGGTAATACTCGCCAG
Mus *Igfbp5*	Forward primer	AGGTGTGGCACTGAAAGTC
	Reverse primer	TGTGACCGCAAAGGATTCTAC
Mus *Il18r1*	Forward primer	CTCGCCCAGAGTCACTTTTCA
	Reverse primer	AGAAATGTACGTTCCCTCATCCT
Mus *Pdk4*	Forward primer	CCGCTTAGTGAACACTCCTTC
	Reverse primer	TGACCAGCGTGTCTACAAACT
Homo *ACTB*	Forward primer	CATGTACGTTGCTATCCAGGC
	Reverse primer	CTCCTTAATGTCACGCACGAT
Mus *Actb*	Forward primer	GTCGTACCACAGGCATTGTGATGG
	Reverse primer	GCAATGCCTGGGTACATGGTGG
